# Kataegis in clinical and molecular subgroups of primary breast cancer

**DOI:** 10.1038/s41523-024-00640-8

**Published:** 2024-04-24

**Authors:** Srinivas Veerla, Johan Staaf

**Affiliations:** Division of Translational Cancer Research, Department of Laboratory Medicine, Lund University, Lund, Sweden

**Keywords:** Breast cancer, Cancer genetics, Cancer genomics, Prognostic markers

## Abstract

Kataegis is a hypermutation phenomenon characterized by localized clusters of single base pair substitution (SBS) reported in multiple cancer types. Despite a high frequency in breast cancer, large-scale analyses of kataegis patterns and associations with clinicopathological and molecular variables in established breast cancer subgroups are lacking. Therefore, WGS profiled primary breast cancers (*n* = 791) with associated clinical and molecular data layers, like RNA-sequencing data, were analyzed for kataegis frequency, recurrence, and associations with genomic contexts and functional elements, transcriptional patterns, driver alterations, homologous recombination deficiency (HRD), and prognosis in tumor subgroups defined by ER, PR, and *HER2*/*ERBB2* status. Kataegis frequency was highest in the HER2-positive(p) subgroups, including both ER-negative(n)/positive(p) tumors (ERnHER2p/ERpHER2p). In TNBC, kataegis was neither associated with PAM50 nor TNBC mRNA subtypes nor with distant relapse in chemotherapy-treated patients. In ERpHER2n tumors, kataegis was associated with aggressive characteristics, including PR-negativity, molecular Luminal B subtype, higher mutational burden, higher grade, and expression of proliferation-associated genes. Recurrent kataegis loci frequently targeted regions commonly amplified in ER-positive tumors, while few recurrent loci were observed in TNBC. SBSs in kataegis loci appeared enriched in regions of open chromatin. Kataegis status was not associated with HRD in any subgroup or with distinct transcriptional patterns in unsupervised or supervised analysis. In summary, kataegis is a common hypermutation phenomenon in established breast cancer subgroups, particularly in HER2p subgroups, coinciding with an aggressive tumor phenotype in ERpHER2n disease. In TNBC, the molecular implications and associations of kataegis are less clear, including its prognostic value.

## Introduction

Breast cancer genomes are shaped by somatic changes, including epigenetic and DNA alterations like single base pair substitutions (SBSs), indels, structural rearrangements, and copy number alterations (CNAs), which together infer high molecular heterogeneity even across patients with similar clinical features. The activity of several mutational processes has been demonstrated in breast cancer, including endogenous processes like DNA repair deficiency and APOBEC mutagenesis^1^. The genetic readout of many mutational processes can be approximated through the concept of mutational signatures, as illustrated by Alexandrov et al. in 2013 for mutational SBS signatures^2^. Currently, a variety of different SBS signatures, indel signatures, structural rearrangement signatures, and CNA signatures have been reported^1–5^. The most studied type of mutational signatures is the SBS signatures which are based on the trinucleotide context of SBSs, i.e., the triplet of bases comprising the single base alteration and adjacent bases immediately 5’ and 3’^3^. Currently, 49 different SBS signatures have been reported based on pan-cancer analysis^5^.

Two of the most distinct SBS signatures with respect to their trinucleotide contexts are signatures SBS2 and SBS13. These signatures were identified already in the breast cancer study by Nik-Zainal et al.^3^ and have been associated with the activity of APOBEC cytidine deaminases (APOBEC mutagenesis)^2^. APOBEC mutagenesis has, in turn, been linked to a mutational phenomenon referred to as kataegis, which is characterized by clusters of SBS hypermutation biased towards a single DNA strand shown to co-localize with specific rearrangement signatures (typically signatures 4 and 6) at the vicinity of structural rearrangements, specifically those in the 10–25 kilobase (kb) range^2–4,6^. Kataegis hypermutation typically comprises C>N mutations in a TpC context^1^, although a T>N conversion in a TpT or CpT process attributed to error-prone polymerases has also been reported^7^ as well as rare occurrences of an alternative kataegis form with a base substitution pattern most closely matching SBS signature 9^4^. Kataegis is proposed to be due to the dominant acting apolipoprotein B editing catalytic subunit 3b (*APOBEC3B*) enzyme that deaminates genomic DNA cytosines and promotes mutation rates higher than normal^8^. Kataegis is typically defined by an intermutation distance between adjacent SBSs, e.g., as six or more consecutive mutations with an average intermutation distance of ≤1000 bp^2^. In a recent pan-cancer whole genome sequence (WGS) based study, kataegis events were found in 60.5% of all cancers, with particularly high abundance in lung squamous cell carcinoma, bladder cancer, acral melanoma, and sarcomas^6^. The APOBEC signature accounted for 81.7% of these kataegis events, while 5.7% of kataegis events involved the T>N error-prone polymerase signature, and 2.3% of events showed cytidine deamination in an alternative GpC or CpC context^6^.

In breast cancer, kataegis has been reported in up to 50% of tumors^3,4,9^, often targeting tumors with high exposure to rearrangement signatures 4 and 6 but not cancers with tandem duplications or deletions of rearrangement signatures 1, 3, and 54. Despite this high frequency, there is a shortage of studies that have analyzed the frequency and associations of kataegis with clinicopathological and molecular variables in large patient cohorts stratified by established clinical and molecular subgroups. In 2016, D’ Antonio et al.^9^ reported the analysis of a cohort of 97 breast cancers using WGS. This study reported that kataegis was associated with a distinct transcriptional signature, late-onset disease, better patient prognosis, and higher HER2 levels. Additionally, it revealed an enrichment of kataegis events on chromosomes 8, 17, and 22 and a depletion of these events on chromosomes 2, 9, and 16. However, many of the significant clinical and molecular associations reported in that study were based on the usage of a transcriptional classifier of kataegis in a large gene expression cohort lacking WGS data (The METABRIC cohort^10^). Thus, kataegis, as defined by WGS (the gold standard), still appears remarkedly understudied in established clinical and molecular subgroups of breast cancer.

To address the limited understanding of kataegis in early-stage primary breast cancer, our study aimed to comprehensively describe, characterize, and analyze the association of kataegis with clinicopathological and molecular factors, transcriptional patterns, and patient outcomes with a focus on established clinical and molecular subgroups defined by ER, PR, HER2, PAM50, and homologous recombination deficiency (HRD) status. Our analysis is conducted on a set of 791 primary breast cancers profiled by WGS and coupled with additional clinical and molecular data. This undertaking represents a scale of studying kataegis not previously reported.

## Results

### Clinical and molecular characteristics of SCAN-B and BASIS cohorts

Characteristics of the investigated SCAN-B and BASIS cohorts are outlined in Table 1. The SCAN-B and BASIS TNBC cohorts were analyzed separately due to a significant difference in binary kataegis frequency, but also to provide validation of results in independent cohorts. It should be acknowledged that the BASIS cohort is not a population-representative cohort and is underpowered with respect to the analyzed number of HER2-positive tumors.

**Table 1:** Clinicopathological information, molecular subtype, and HRD status per cohort

Feature	SCAN-B TNBC	BASIS TNBC	BASIS ERpHER2n	BASIS HER2-positive^a^
Number of samples with WGS	100% (235)	100% (163)	100% (320)	100% (73)
Number of RNA-sequenced tumors	100% (235)	44.8% (73)	58.1% (186)	5.5% (4)
Clinical subgroups				
TNBC	100% (235)	100% (163)	0% (0)	0% (0)
ER-positive & HER2-negative (ERpHER2n)	0% (0)	0% (0)	100% (320)	0% (0)
ER-positive & HER2-positive (ERpHER2p)	0% (0)	0% (0)	0% (0)	63% (46)
ER-negative & HER2-positive (ERnHER2p)	0% (0)	0% (0)	0% (0)	37% (27)
PAM50 subtypes^b^				
Basal-like	79.9% (187)	83.6% (61)	0.5% (1)	25% (1)
HER2-enriched (HER2E)	14.5% (34)	8.2% (6)	2.2% (4)	0% (0)
Luminal A	2.1% (5)	1.4% (1)	39.2% (73)	25% (1)
Luminal B	0.4% (1)	4.1% (3)	56.5% (105)	50% (2)
Normal-like	3.0% (7)	2.7% (2)	1.6% (3)	0% (0)
HRDetect-low	40.9% (96)	51.5% (84)	91.6% (293)	95.9% (70)
HRDetect-high	59.1% (139)	48.5% (79)	8.4% (27)	4.1% (3)

Values are reported as percentages with numbers in ().

^a^Includes ERnHER2p and ERpHER2p cases.

^b^As reported in original studies based on respective classification method. Percentages are computed excluding any sample with missing data (NAs). Not all BASIS tumors have associated RNA-sequencing data to allow PAM50 classification.

### Frequency of kataegis in clinical and molecular subgroups of breast cancer

**Fig. 1:**
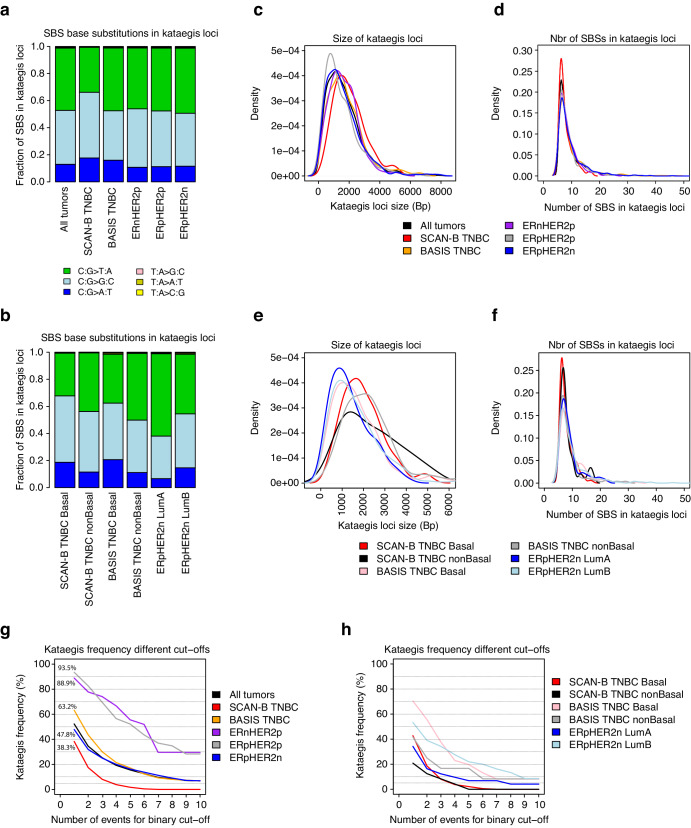
Analyses of SBS patterns, kataegis loci size, and effects of different cut-offs for binary kataegis frequency in clinical and molecular subgroups of breast cancer. **a** Base substitution pattern for SBSs in kataegis loci for tumors in clinical subgroups. All SBSs in kataegis loci in all tumors of a subgroup were merged and analyzed as a single set. **b** Same analysis as in **a**, but for different molecular subgroups. **c** Kataegis loci size in base pairs for clinical subgroups. To facilitate co-plotting, lines represent the smoothed density kernel approximation of underlying histograms. **d** Number of SBSs in kataegis loci size for clinical subgroups. Lines represent smoothed density kernel approximation of underlying histograms to facilitate co-plotting. **e** Same analysis as in **c** but for different molecular subgroups. **f** Same analysis as in **d** but for different molecular subgroups. **g** Effect of different cut-offs (number of kataegis events) for binary kataegis frequency in clinical subgroups. Displayed percentages in the text refer to the frequency for cut-off ≥ 1 kataegis event. **h** Effect of different cut-offs (number of kataegis events) for binary kataegis frequency in molecular subgroups. LumA Luminal A, LumB Luminal B, nonBasal any PAM50 subtype other than Basal.

Across all analyzed tumors 2039 kataegis loci were identified in 413 (52.2%) of 791 tumors. SBSs in kataegis loci showed a high frequency of C>T and C>G substitutions (consistent with previous reports^11^) in both clinical and molecular subgroups of breast cancer (Fig. 1a, b). Moreover, the kataegis loci base pair size and number of SBSs in kataegis loci appeared similar across clinical subgroups (Fig. 1c, d), while some differences appeared for kataegis loci size, e.g., more smaller loci in ERpHER2n Luminal A and Luminal B tumors but not number of SBSs, in molecular subgroups (Fig. 1e, f). It should be noted that the sample sizes for the molecular subgroups in Fig. 1b, e, and f are notably smaller than for the clinical subgroups, which may impact observed patterns.

In a binary context the frequency of kataegis (≥1 event on chromosomes 1–23) was 38.3% in SCAN-B TNBC, 63.2% in BASIS TNBC, 88.9% in BASIS ERnHER2p, 93.5% in BASIS ERpHER2p, and 47.8% in BASIS ERpHER2n (Fig. 1g). Increasing the binary cut-off value (i.e., stringency) for kataegis-positivity reduced the kataegis frequency in both clinical and molecular subgroups (e.g., 15–30% reduction in clinical subgroups with a cut-off ≥3 events) (Fig. 1g, h). Still, the pattern of a higher kataegis frequency in HER2p disease groups remained consistent across cut-offs, as well as the pattern of higher kataegis frequency in ERpHER2n Luminal B tumors compared to Luminal A tumors. It should be noted that there are significantly fewer tumors in several of the molecular subgroups due to incomplete gene expression data for BASIS tumors.

**Fig. 2:**
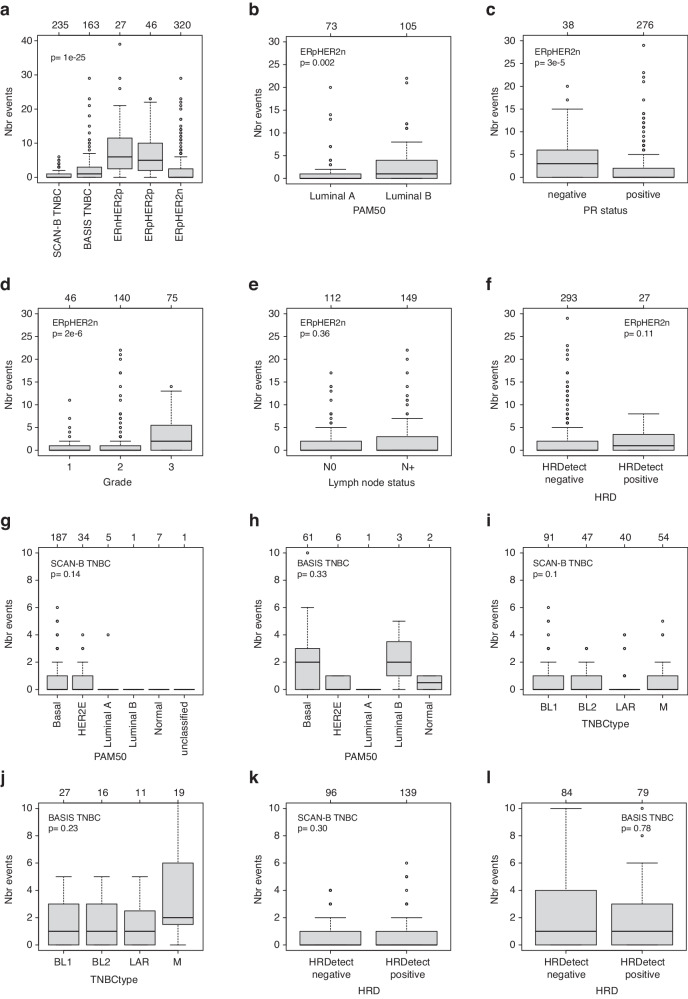
Number of kataegis events in breast cancer subgroups. **a** Clinical subgroups defined by ER, PR, and HER2-status. SCAN-B and BASIS TNBCs are separately displayed. **b** PAM50 Luminal A and Luminal B in ERpHER2n tumors. **c** Progesterone receptor (PR) status in ERpHER2n tumors. **d** Tumor grade in ERpHER2n tumors. **e** Lymph node status in ERpHER2n tumors. **f** HRD status in ERpHER2n tumors. **g** PAM50 subtypes in SCAN-B TNBC tumors. **h** PAM50 subtypes in BASIS TNBC tumors. **i** TNBCtype subtypes in SCAN-B TNBC tumors. **j** TNBCtype subtypes in BASIS TNBC tumors. **k** HRD status in SCAN-B TNBC tumors. **l** HRD status in BASIS TNBC tumors. *P*-values were calculated using Wilcoxon’s test (2-group) or Kruskal–Wallis test (>2 groups). In panels **g**–**l** the *y*-axis is truncated at 10 events for comparative reasons between SCAN-B and BASIS. Boxplot elements correspond to: (i) center line = median, (ii) box limits = upper and lower quartiles, and (iii) whiskers = 1.5× interquartile range.

The number of kataegis events per tumor was investigated in four clinical subgroups of primary breast cancer: TNBC, ERnHER2p, ERpHER2p, and ERpHER2n, demonstrating a markedly higher number of kataegis loci in HER2p groups irrespective of ER-status (Fig. 2a). Next, we analyzed the number of kataegis events in ERpHER2n tumors stratified by PAM50 subtype (restricted to Luminal A versus Luminal B subtypes due to sample sizes), progesterone receptor (PR) status, tumor grade, lymph node status, and HRDetect status (Fig. 2b–f). These analyses demonstrated that kataegis events were more frequent in ERpHER2n PAM50 Luminal B tumors compared to Luminal A tumors, more frequent in PR-negative versus PR-positive tumors, and more frequent in high-grade tumors compared to lower grades, while no differences were observed for lymph node status or HRDetect status. Histological subtype data was available for the BASIS cohort. In ductal cancers, the overall binary kataegis frequency was 62%, and in lobular cancers, 44%. In ERpHER2n tumors, the corresponding frequencies were 53% and 39%, respectively.

While lower sample numbers precluded subgroup analyses within the ERnHER2p and ERpHER2p groups, we could analyze kataegis in both SCAN-B (*n* = 235) and BASIS TNBC (*n* = 163) tumors similar to ERpHER2n tumors. These analyses were performed separately for each TNBC cohort as we did observe a significant difference in binary kataegis frequency between the two cohorts (Chi-square test *p* = 2e−6). In neither SCAN-B nor BASIS TNBC tumors were the number of kataegis events associated with PAM50 subtypes, TNBCtype subtypes, HRDetect status, or lymph node status (Fig. 2g–l and Supplementary Fig. 1). Grade 3 SCAN-B TNBC tumors showed more kataegis events compared to lower stages (Kruskal–Wallis *p* = 0.01), but this observation was not supported in BASIS TNBC tumors (*p* = 0.24) (Supplementary Fig. 1). In the set of 791 analyzed tumors, 11 tumors have been reported as mismatch repair deficient (MMRd)^12,13^, of which four (30.8%) had a kataegis event (Chi-square test *p* = 0.20).

### Association of kataegis with patient age and molecular variables in breast cancer subgroups

**Fig. 3:**
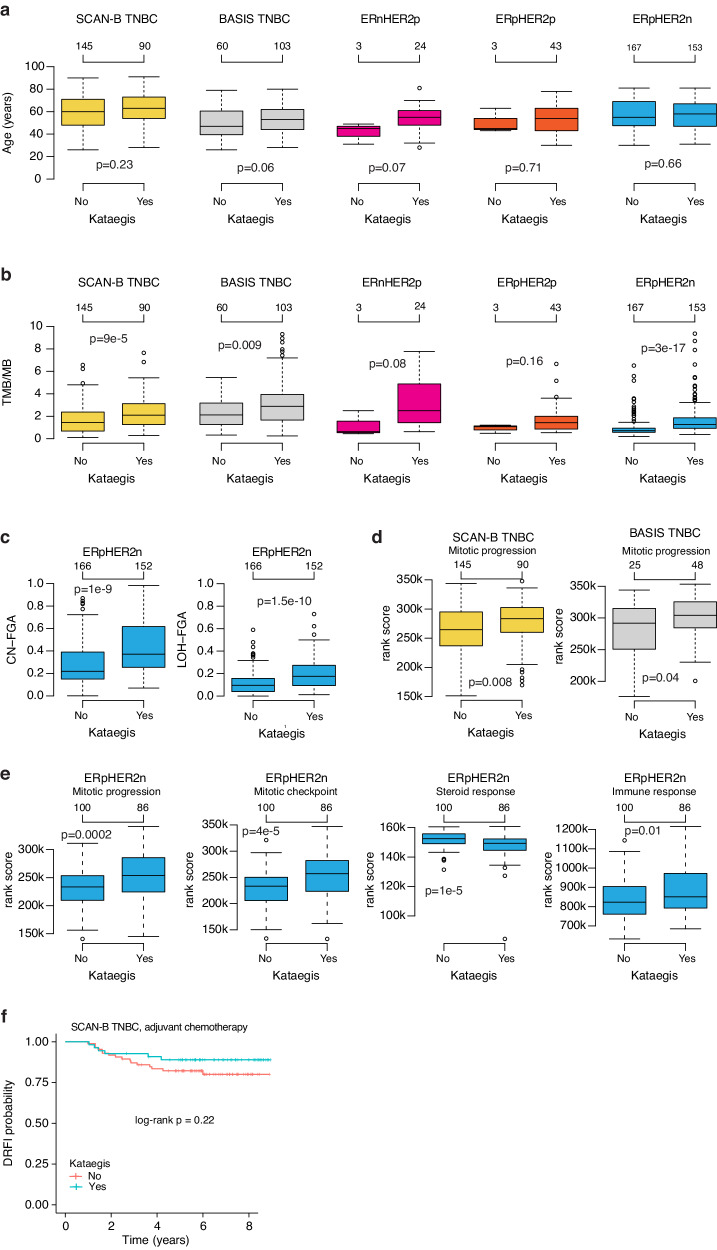
Associations of kataegis with patient age, molecular variables, and patient outcome in breast cancer subgroups. **a** Distribution of patient age versus binary kataegis classification in breast cancer subgroups defined by ER, PR, and HER2. **b** Distribution of tumor mutational burden (sum of SBS and indels per million base pair sequence) versus binary kataegis classification in ER, PR, and HER2-defined subgroups. **c** Distribution of fraction of the genome altered by copy number gain or loss (CN-FGA, top) or affected by LOH (bottom) versus binary kataegis classification in ERpHER2n tumors. Two tumors were excluded due to failed analysis. A value of 0 means that no parts of the genome (chromosomes 1–22) are affected by somatic changes, and a value of 1 that the entire tumor genome is affected. **d** Mitotic progression metagene^14^ rank scores for SCAN-B and BASIS TNBC tumors stratified by binary kataegis status. **e** Rank scores for four biological metagenes from Fredlund et al.^14^ showing a difference in scores for binary kataegis status in ERpHER2n tumors. **f** Kaplan–Meier plot of the association of binary kataegis status with distant relapse-free interval (DRFI) as a clinical endpoint in SCAN-B TNBC patients treated with adjuvant chemotherapy. The P-value was calculated using the log-rank test. All BASIS tumors do not have matched RNA-sequencing data. *P*-values were calculated using Wilcoxon’s test (2-group) or Kruskal–Wallis test (>2 groups). Boxplot elements correspond to: (i) center line = median, (ii) box limits = upper and lower quartiles, (iii) whiskers = 1.5× interquartile range.

Kataegis has been proposed to be associated with a higher age at diagnosis of breast cancer^9^. In SCAN-B TNBC, BASIS TNBC, ERpHER2p, and ERpHER2n tumors, binary kataegis was not significantly associated with patient age (Wilcoxon’s test *p* > 0.05) (Fig. 3a). Only in the smallest clinical group, ERnHER2p (*n* = 27 tumors), was a notably higher age at diagnosis in kataegis positive (≥1 kataegis event) patients observed, albeit not statistically significant (Wilcoxon’s test *p* = 0.07) and based on small patient numbers (Fig. 3a).

To explore associations of kataegis with genomic variables, we analyzed if binary kataegis status was associated with tumor ploidy, mutational burden, the fraction of the genome altered by copy number alterations, and the fraction of the genome affected by LOH in TNBC, ERnHER2p, ERpHER2p, and ERpHER2n tumors. A significant association or trend of higher tumor mutational burden (TMB) with positive kataegis status was observed across all subgroups (Fig. 3b). For tumor ploidy, higher ploidy was observed in kataegis positive BASIS TNBC (Wilcoxon’s test *p* = 0.04), but not in SCAN-B TNBC or any other tested subgroup (Supplementary Fig. 1). For both the fraction of the genome affected by copy number alterations or by LOH, significantly higher fractions (i.e., a more altered tumor genome) were only observed in kataegis-positive ERpHER2n tumors (Fig. 3c and Supplementary Fig. 1). The above-described associations for TMB, LOH, and amount of copy number alterations remained significant also when changing the binary kataegis threshold to ≥2 events or ≥3 events for the specific comparisons (Supplementary Fig. 1).

To investigate if kataegis was associated with broader transcriptional programs, we analyzed expression rank scores of eight biological metagenes (from ref. ^14^) in the clinical subgroups stratified by binary kataegis status. Due to a lack of matched gene expression data for BASIS HER2-positive tumors, this analysis was restricted to the TNBC and ERpHER2n groups. In both TNBC cohorts, this analysis identified only a weaker increase in metagene rank scores for the mitotic progression (proliferation-associated) metagene in kataegis-positive tumors (Wilcoxon’s test *p* < 0.05 in both SCAN-B and BASIS) (Fig. 3d). When increasing the binary cut-off to ≥2 events the statistical significance was lost in the BASIS TNBC cohort, suggesting that the biological association is of weaker nature. In BASIS TNBCs, kataegis-positive tumors showed lower rank scores of the immune response metagene (Wilcoxon’s test *p* < 0.05), with a similar, but non-significant, trend of lower immune response rank scores observed also in kataegis-positive SCAN-B tumors (Wilcoxon’s test *p* = 0.15). Moreover, a non-significant trend of lower TIL scores was also observed for kataegis-positive TNBC tumors in the SCAN-B cohort (Wilcoxon’s test *p* = 0.16). In ERpHER2n tumors, several metagenes showed significant rank score differences between kataegis positive and negative cases, including higher rank scores of the mitotic progression and mitotic checkpoint metagenes (both associated with expression of proliferation-related genes), as well as higher immune response scores in the kataegis-positive group (Fig. 3e). In contrast, rank scores for the steroid response metagene were lower in kataegis positive ERpHER2n tumors (Fig. 3e). The associations for the immune response, mitotic progression, mitotic checkpoint, and steroid response metagenes remained significant also when changing the binary kataegis threshold to ≥2 events and ≥3 events in ERpHER2n tumors (Supplementary Fig. 1).

### Association of kataegis with driver alterations in breast cancer subgroups

To investigate if specific tumor driver alterations were associated with binary kataegis status in the clinical subgroups, we analyzed driver gene alterations (SBS mutations, indels, structural rearrangements, and copy number alterations) based on reported driver events obtained from the original studies^4,13^. In ERnHER2p tumors, no significant associations were identified, potentially because of the small group size (Chi-square test *p* > 0.05). In the small ERpHER2p subgroup, a difference in *CDH1* alterations was found (Chi-square test *p* = 0.02, FDR adjusted *p* = 0.68) with a higher frequency in kataegis negative tumors. This observation is consistent with an overall lower binary kataegis frequency in lobular BASIS cancers with *CDH1* alterations (33%) compared to lobular cancers without *CDH1* alterations (57%), albeit not reaching statistical significance due to small sample groups (Chi-square test *p* = 0.32). *PIK3CA* and *PIK3R1* alterations were more frequent in kataegis-negative BASIS TNBC tumors (*p* = 0.01 and *p* = 0.02, respectively), but p-values were non-significant after FDR adjustment. In line with the latter, neither *PIK3CA* nor *PIK3R1* showed significant associations with kataegis in the SCAN-B cohort. In contrast, in the SCAN-B TNBC cohort only alterations involving *CCND1*, *KRAS*, *ARID1B*, and *TP53* were found to be more frequent in kataegis-positive tumors (Chi-square test *p* < 0.05), although only *TP53* remained significant after FDR adjustment (FDR *p* = 0.03). In ERpHER2n tumors, alterations involving *CCND1* (11q13.3), *ZNF703*/*FGFR1* at 8p12, *MDM2* (12q15), *MYC* (8q24.21), *TP53*, and *ZNF217* (20q13.2) were all more frequent in kataegis positive tumors, while *PIK3CA* and *MAP3K1* alterations were more frequent in kataegis negative tumors (Chi-square test *p* < 0.05). After FDR correction for multiple tests, *MDM2* and *MAP3K1* alterations did not reach statistical significance (FDR *p* > 0.05).

### Association of kataegis with patient outcome after adjuvant chemotherapy in TNBC

Association of binary kataegis status with DRFI after adjuvant chemotherapy (mainly FEC-based therapy, see ref. ^13^) was investigated in SCAN-B TNBC patients, however, without finding any significant association (Fig. 3f). To assure that the binary stratification based on detecting at least one event did not bias the survival analysis, we also conducted survival analysis using two and three events as cutoffs. No significant association was found in these analyses (log-rank *p* > 0.05). Due to incomplete survival and treatment data, similar survival analyses could not be performed in the TNBC, ERpHER2n, ERnHER2p, or ERpHER2p subgroups within the BASIS cohort.

### Genomic contexts of kataegis loci in clinical breast cancer subgroups

**Fig. 4:**
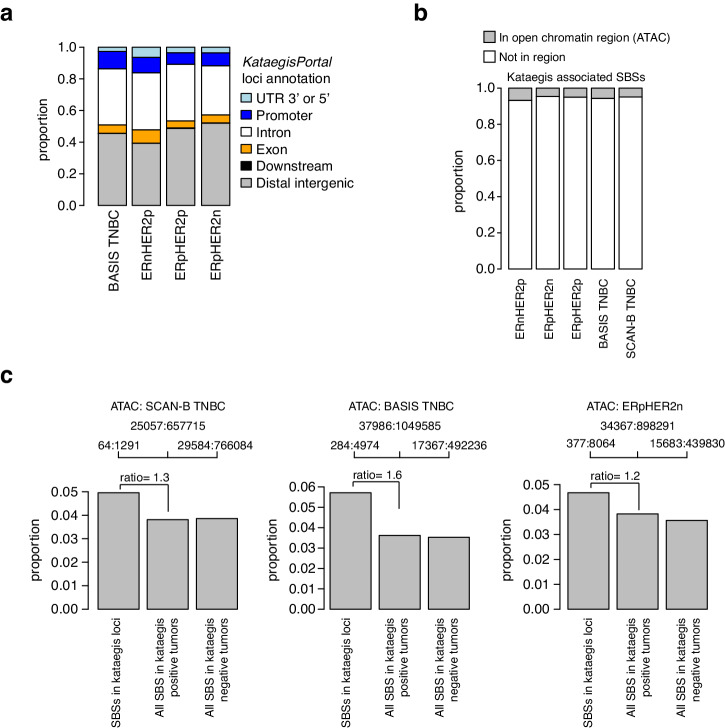
Genomic contexts of kataegis in clinical breast cancer subgroups. **a** Proportion of all kataegis events (loci) in a clinical subgroup in the BASIS cohort annotated to different genomic contexts by the *KataegisPortal* tool. Detailed contexts from the tool have been aggregated to the specified larger annotations for each locus. **b** Proportion of kataegis-associated SBSs (i.e., SBSs in a kataegis loci) mapped to regions of open chromatin (ATAC) per clinical subgroup. For each subgroup, all SBSs associated with kataegis that were mapped or not mapped to ATAC regions were summarized across all tumors, i.e., proportions represent the proportion of all kataegis SBS mapped or not in kataegis-affected tumors only. **c** Proportions of ATAC mapped kataegis SBS from panel **b** compared to similarly computed proportions based on all SBSs in kataegis positive and negative tumors for the SCAN-B TNBC, BASIS TNBC, and ERpHER2n subgroups. Top axis for each bar reports the number of SBSs mapped to ATAC across all tumors and the total number of SBS across all tumors in the group. The displayed ratio corresponds to the kataegis SBS bar divided by the “all SBS” bar for kataegis-positive tumors.

To investigate the contexts of kataegis loci, we aggregated the *KataegisPortal* annotations for each kataegis loci for all kataegis events in a clinical subgroup and compared proportions between subgroups. The subgroup comparison was restricted to the BASIS cohort and showed similar distributions of kataegis loci (Fig. 4a), with a majority of loci annotated to be in distal intergenic (39-52%) or intronic regions (31–36%), while ~7–10% of events were in promoter regions. In comparison, in the SCAN-B TNBC cohort, 48% of kataegis events were in distal intergenic regions, 40% in intronic regions, while 7% in promoter regions.

We further examined the distribution of SBSs associated with kataegis events across the genome with respect to different genomic contexts and functional elements for each clinical subgroup (excluding the HER2p groups due to small sample sizes). To this end, each SBS involved in a kataegis event was annotated by mapping to regions of open chromatin (based on ATAC-seq data), DHS, intronic, exonic, intergenic, and different repeat regions, as well as functional elements like CTCF binding regions and different chromatin states. For each clinical subgroup, we computed the sum of such SBSs annotated to a specific genomic context, normalized the sum by the total number, and compared the proportions across subgroups. Accordingly, in this comparison only kataegis positive tumors were included. Notably, while proportions differed between investigated contexts, each such pattern was relatively consistent across subgroups (Fig. 4b showing the open chromatin comparison and Supplementary Fig. 2 for all comparisons).

To investigate the enrichment for kataegis involvement among SBSs in various genomic contexts we extended our analysis to include both kataegis positive and kataegis negative tumors. We computed the proportions of SBSs using an approach analog to that illustrated in Fig. 4b, now applying it to all SBSs. This analysis was restricted to TNBC and ERpHER2n due to sample sizes. Comparisons across subgroups identified higher proportions of kataegis-associated SBSs mapped to open chromatin regions versus the full SBS context in both kataegis-positive and negative tumors (Fig. 4c and Supplementary Fig. 3 for all comparisons). Similarly, considerably weaker positive trends were also observed for DHS regions and intronic regions. Weak opposite trends (i.e., lower proportions for kataegis-associated SBSs versus an all-SBS context) were observed for SINE and low complexity repeat elements. Using this analysis approach, we found no evidence of enrichment or depletion of kataegis-associated SBSs for chromatin states related to transcription start site (TSS) elements, different enhancer elements, or quiescent chromatin regions (loci not transcribed) compared to the full SBS context in both kataegis positive and negative tumors (Supplementary Fig. 3).

### Patterns of recurrent kataegis in clinical breast cancer subgroups

**Fig. 5:**
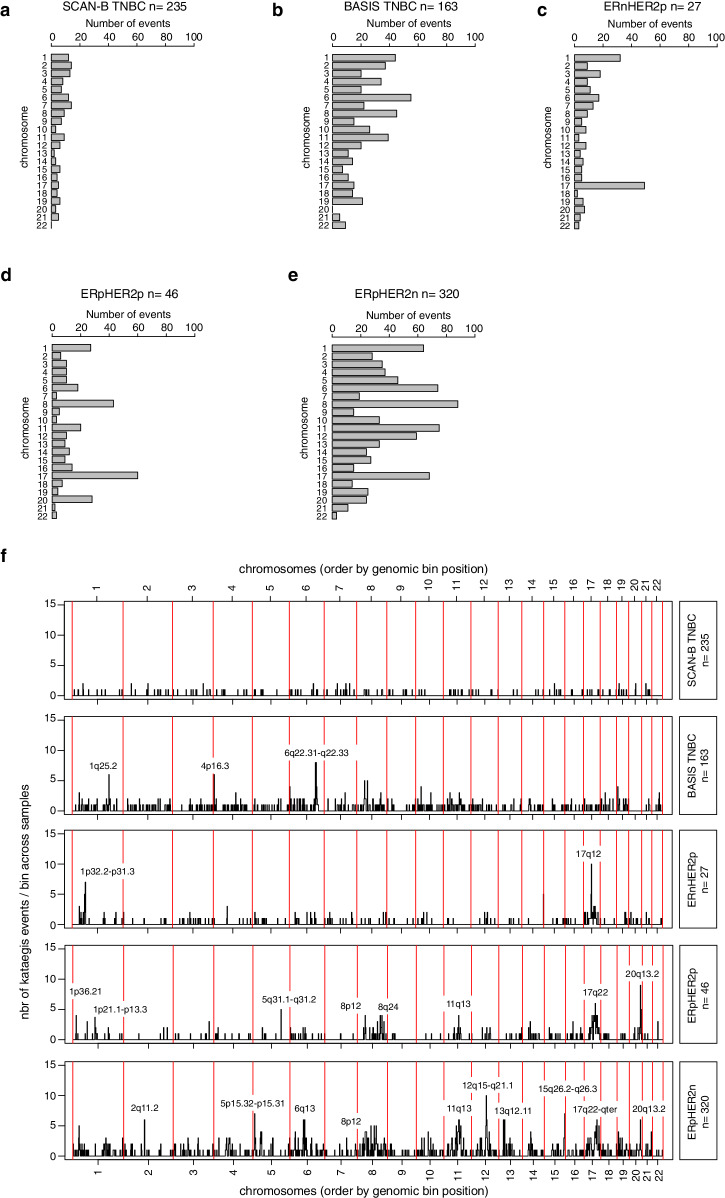
Genome patterns of recurrent kataegis in clinical breast cancer subgroups. Panels **a**–**e** show the number of kataegis events per chromosome (chromosome 1–22) across all tumors in a subgroup. **a** SCAN-B TNBC, **b** BASIS TNBC, **c** ERnHER2p, **d** ERpHER2p, and **e** ERpHER2n. **f** Genome-wide frequency plots of the number of mapped kataegis events per 2MBp bin (*y*-axis) per tumor subgroup, ordered according to genomic position (*x*-axis). Technically, multiple kataegis loci could be mapped to the same bin if occurring close to each other. Chromosome boundaries are indicated by vertical red lines. Bins with recurrent kataegis events are labeled by cytoband.

To coarsely illustrate the prevalence of kataegis loci across the genome, we compared the number of events detected per chromosome and clinical tumor subgroup (Fig. 5a–e). This analysis revealed subgroup differences regarding chromosomes frequently harboring kataegis events. For instance, chromosomes 17 and 1 frequently harbored kataegis events in all subgroups except TNBC. Events appeared more often on chromosome 8 in ER-positive subgroups compared to ER-negative tumors, whereas chromosomes 6, 11, and 12 harbored events more often in ERpHER2n tumors compared to the other subgroups. It should be noted that sample numbers are small for the HER2p groups so there is uncertainty in conclusions for these subgroups. Moreover, the chromosome analysis also demonstrated a difference between the two TNBC cohorts, supporting that they should be examined separately.

To expand our analysis of the recurrence of kataegis events across the genome from Fig. 5a–e, we next increased the analysis resolution beyond whole chromosomes by counting events for each tumor mapped to consecutive 2 MBp-sized bins throughout the genome (i.e., bins can include multiple closely spaced kataegis events for a single sample). Using these counts, we then generated frequency plots for the respective tumor subgroups (Fig. 5f and Supplementary Table 1 for detailed frequency data). This analysis demonstrated reoccurring events in regions closely adjacent to or coinciding with well-established copy number amplification loci, particularly within ERpHER2n tumors, such as 11q13 (*CCND1*), 8p12 (*FGFR1*/*ZNF703*), 12q15-q21.1 (*MDM2*), several regions at 17q22-qter, and 20q13.2 (*ZNF217*), but also additional small regions at 2q11.2, 5p15.32-p15.31, 6q13, 13q12.11, 13q12.13-q12.2, and 15q26.2-q26.3 (including *IGF1R*). While recurrent, event frequency (i.e., the number of events in Fig. 5f/number of tumors per group) for genomic location was still <5% for the most recurrent bins. In SCAN-B TNBC tumors, there were no obvious recurrent kataegis events at all, and in BASIS TNBC tumors only regions on 1q25.2 (including, e.g., *ABL2*), 4p16.3 (including, e.g., *POLN*), and 6q22.31-q22.33 (including, e.g., *CENPW*, *THEMIS*, and *PTPRK*) appeared as recurrent. In ERnHER2p tumors, the most recurrent region was at 17q12 (including *ERBB2*), and in addition also, a small region on 1p32.2-p31.3 (including e.g., *JUN*, *FOXD3*, and *ITGB3BP*). In ERpHER2p tumors the most recurrent regions overlapped with or were in close proximity to typical sites of recurrent amplification in breast cancer such as 8p12, 8q24, 11q13, 20q13.2 (*ZNF217*), 17q12 (including *ERBB2*), 17q22, but also small regions on 5q31.1-q31.2 (e.g., *BRD8*, *CDC23*), 1p21.1-p13.3, and 1p36.21 (see Supplementary Table 1 for details and gene lists).

### Transcriptional patterns associated with kataegis in breast cancer subgroups

**Fig. 6:**
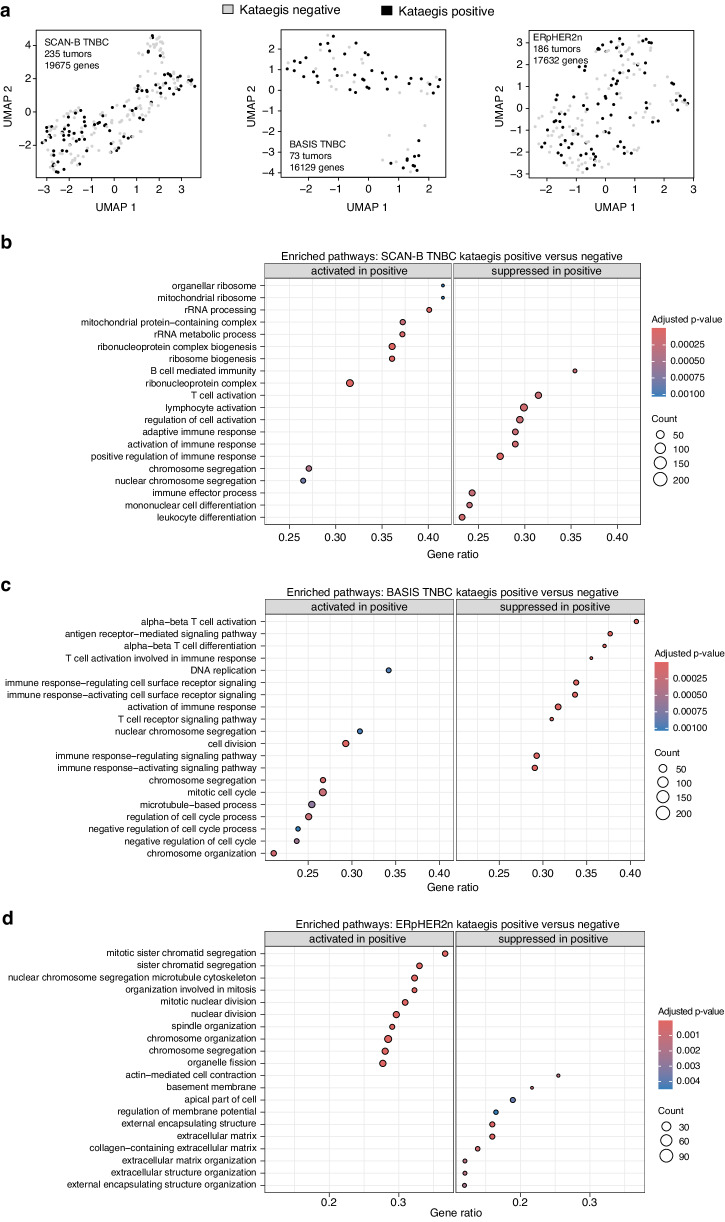
Unsupervised and supervised gene expression patterns associated with kataegis in clinical breast cancer subgroups. **a** UMAP analysis of FPKM data from SCAN-B TNBC (left: 235 tumors, 19,675 genes), BASIS TNBC (center: 73 tumors, 16,129 genes), and ERpHER2n (right: 186 tumors, 17,632 genes). Tumors are colored by binary kataegis event status (positive: ≥1 event, negative: 0 events). The first two UMAP components are shown. Only genes with expression for all samples in a group are included in the analyses, thereby, the different numbers for the two BASIS cohorts. **b** GSEA analysis for significantly activated and suppressed pathways in SCAN-B TNBC tumors stratified into kataegis positive or negative as in (**a**). **c** GSEA analysis for significantly activated and suppressed pathways in BASIS TNBC tumors stratified into kataegis positive or negative as in (**a**). **d** GSEA analysis for significantly activated and suppressed pathways in ERpHER2p tumors stratified into kataegis positive or negative as in (**a**). In **b**–**d**, the Gene ratio axis represents the number of genes from the query gene list that overlap with the gene set of a specific pathway or Gene Ontology (GO) term. This ratio is calculated by dividing the number of overlapping genes by the total number of genes in the set. The count circle size represents the number of query gene lists that overlap with the gene set and the adjusted *p*-value represents the statistical significance of the enrichment, adjusted for multiple testing using Benjamini–Hochberg (BH) correction.

To investigate if binary kataegis status could explain general transcriptional variation in breast cancer, we first performed an unsupervised UMAP clustering of FPKM data in the SCAN-B TNBC, BASIS TNBC, and ERpHER2n subgroups based on available matched expression data (ERnHER2p and ERpHER2p excluded due to lack of matched RNA-sequencing data in BASIS). As shown in Fig. 6a, UMAP analysis provided no apparent evidence of association with global gene expression patterns.

Next, we performed supervised SAM analysis within the clinical subgroups to identify differentially expressed genes (DEGs) between kataegis positive and negative tumors. This analysis identified 2148 DEGs in SCAN-B TNBC, 882 in BASIS TNBC, and 1976 in ERpHER2n tumors (unadjusted SAM *p* < 0.05). After multiple correction adjustments, no genes remained significant in the TNBC cohorts, and only three genes (*SIAE*, *LIMCH1*, and *NTN4*) remained significant in ERpHER2n tumors (adjusted *q* < 0.05). The near absence of significant DEGs after multiple testing corrections contrasts with the previously reported number of 628 genes identified as differentially expressed^9^. This discrepancy prompted us to investigate the expression patterns of these genes in the TNBC cohorts and ERpHER2n tumors analyzed in our study. In the SCAN-B TNBC cohort, four of the 556 matched genes had a significant FDR-adjusted *p*-value based on an unpaired t-test analysis (*p* < 0.05); however, all four genes had median FPKM values in both the kataegis positive and negative groups below 1.6 (i.e., very low expression), indicating that the significance was driven by outliers. For BASIS TNBC, the corresponding numbers were zero significant genes out of 183 genes with complete log2 transformed FPKM data for all tumors (i.e., no missing values). For the ERpHER2n group, 14 out of 202 matched genes were significant (FDR-adjusted *p* < 0.05). These 14 genes were *FGF10*, *QSOX1*, *PLEK2*, *RGS5*, *GLRB*, *AIM1*, *WHSC1L1*, *PRR11*, *ENTPD5*, *TUBA3D*, *BRF2*, *CX3CR1*, *RAB3D*, and *TMEM132A*.

Given the predominantly negative results in our supervised analysis of DEGs, we proceeded with a GSEA analysis to explore gene sets distinguishing between kataegis positive and negative tumors. This analysis identified both activated and suppressed pathways, much in agreement with the biological metagene expression analyses previously shown in Fig. 3. Specifically, in both SCAN-B and BASIS TNBC cases, suppressed GSEA pathways in kataegis positive tumors included different immune pathways, while for ERpHER2n tumors cell cycle/proliferation-associated pathways were activated and pathways associated with extracellular matrix terms were found to be repressed (Fig. 6b–d).

## Discussion

Previous studies have thoroughly investigated the genetic nature and associations of kataegis as a hypermutation event with mutational signatures and structural rearrangements^2,4,6^. In this study, we aimed to investigate the genome-wide patterns, frequency, and associations of kataegis with clinicopathological characteristics, molecular variables, and transcriptional patterns across established clinical and molecular breast cancer subgroups. Although the high frequency of kataegis in breast cancer has been observed repeatedly for over a decade, our study represents a large-scale analysis of the phenomenon that has not been reported to date.

Under a binary categorization, we observed that kataegis frequency was markedly higher in the HER2p subgroups across different binary cut-offs, even though these subgroups had relatively small patient numbers. This observation is consistent with the report by D’ Antonio et al.^9^, as well as a reported higher APOBEC exposure (associated with kataegis) in HER2p breast cancer groups^15,16^. For the HER2p groups, the high kataegis frequency appears to be largely driven by a notably high kataegis frequency on specific chromosomes such as 1, 8, 17 (often including the *ERBB2* locus), and 20 (Fig. 5). A notable observation is the difference (for unknown reasons) in kataegis frequency between the SCAN-B and BASIS TNBC cohorts. This discrepancy underscores the importance of basing conclusions on data from several independent cohorts. In this context, the SCAN-B cohort has been demonstrated to be representative of the underlying TNBC patient population in the catchment region for years of inclusion^13^. This contrasts with the BASIS cohort, which is multi-national and multi-institutional. In ERpHER2n tumors, our combined results show a strong association between kataegis and a typically aggressive ERpHER2n phenotype, including the Luminal B PAM50 subtype, higher tumor grade, PR negativity, higher tumor mutational burden, generally more copy number alterations and LOH, consistent with^9^.

To explore associations of kataegis in clinical breast cancer subgroups, we used available clinical and molecular data from both WGS and RNA sequencing. In contrast to D’ Antonio et al.^9^, we did not find support for kataegis being associated with a higher age at diagnosis in any of the tested clinical subgroups. However, two consistent molecular findings across all subgroups emerged: a significant pattern or trend indicating higher tumor mutational burden in kataegis-positive tumors and a non-significant association of kataegis with HRD status (which is also associated with higher tumor mutational burden). Although higher exposure to APOBEC mutagenesis (associated with kataegis) has been previously reported in HER2p breast cancer groups based on mutational signature analysis15,16, many HRD-positive tumors, e.g., among TNBCs, also show exposure to APOBEC-related mutational signatures like SBS2 and SBS13 (see Supplementary Fig. 4). Given this context, the non-overlapping agreement between two common genetic phenotypes (HRD and kataegis), both of which are driven by endogenous mutational processes, deserves further investigation.

In TNBC, where our insights are more validated based on the usage of two independent cohorts, we found that kataegis was not associated with PAM50 subtypes or proposed TNBC-specific subtypes. Moreover, for adjuvant chemotherapy-treated SCAN-B TNBC patients, kataegis showed no prognostic association with DRFI. This finding aligns logically with the observed insignificant association with HRD status, which has been shown to be prognostic in the SCAN-B cohort^13^. Also, there was no significant association between kataegis and an immune response mRNA metagene or pathology-estimated TIL scores in the SCAN-B cohort, both recognized as prognostic variables in TNBC (see, for example, ref. ^17^).

The driver gene association analysis clearly demonstrated the impact of different cohorts on the results. In ERpHER2n tumors, driver gene alterations associated with a positive kataegis status predominantly included *TP53* and genes in regions commonly amplified in breast cancer. In contrast, kataegis-negative tumors showed more enrichment for PIK3CA alterations. The SCAN-B cohort supported a higher frequency of TP53 alterations in kataegis-positive tumors, a finding not mirrored in the BASIS TNBC cohort. Taken together, the driver gene analysis was most consistent for ERpHER2n tumors, indicating that observed kataegis is associated with recurrent amplifications of key oncogenes in breast cancer, often found in PAM50 Luminal B and HER2p tumors^18,19^.

Kataegis loci have been proposed to stabilize the expression of neighboring genes, and kataegis as a binary event has been reported to be associated with a specific 628 gene mRNA signature based on analysis of 97 tumors of mixed clinical subgroups^9^. In our study, we explored transcriptional patterns associated with kataegis within each clinical subgroup, employing both unsupervised and supervised approaches. Notably, our unsupervised global UMAP analysis demonstrated that binary kataegis status does not appear to significantly explain transcriptional variation in either TNBC or ERpHER2n tumors, suggesting the absence of a distinct transcriptional phenotype attributed to kataegis in these subgroups. Our unsupervised biological metagene analyses indicated that kataegis-positive tumors generally tended to exhibit higher expression of genes associated with proliferation. In ERpHER2n tumors, there was also a trend toward lower expression of steroid response-associated genes. This latter finding is in agreement with a generally lower ER signaling, PR negativity, and a Luminal B subtype, as opposed to the typical patterns in Luminal A tumors (see e.g., data from refs. ^14,20^). A notable distinction between TNBC and ERpHER2n tumors was an inversed pattern of the Fredlund et al.^14^ immune response metagene. In kataegis positive ERpHER2n tumors, there were higher rank scores, whereas in TNBC tumors, there was a trend of lower scores (as well as lower TIL estimates), though this trend was not significant in either BASIS or SCAN-B. Supervised differential gene expression failed to identify significant numbers of DEGs after multiple testing corrections in both TNBC and ERpHER2n tumors. Similarly, the previously reported list of 628 kataegis-associated DEGs in breast cancer^9^ validated poorly in both TNBC cohorts as well as in the ERpHER2n subgroup. Together, these findings support the UMAP results, suggesting that kataegis does not constitute a distinct transcriptional entity.

In contrast with the SAM analyses, the GSEA analysis identified enriched pathways consistent with results from the biological metagene analysis. Specifically, for TNBC, we observed suppressed activity of immune response pathways in both the BASIS and SCAN-B cohorts, while results for activated pathways appeared more mixed. This variability underscores the critical importance of careful curation and control of study cohort composition to draw accurate biological conclusions. For the ERpHER2n subgroup, GSEA analysis confirmed the activation of cell cycle-related pathways in kataegis-positive tumors and reinforced the association of kataegis with an aggressive, typically poor patient outcome phenotype by demonstrating suppression of different ECM-related pathways typically associated with higher metastatic potential. Due to the lack of matched tissue for in situ analyses in our study, it was not possible to address the observed trends regarding the interplay between immune response and kataegis status, a topic that warrants further investigation. Specifically, it remains unclear whether kataegis hypermutation events induce immunogenicity in some molecular background (like ERpHER2n), but not in others (e.g., TNBC), or whether observed associations are more related to other correlative characteristics like a high tumor mutational burden and frequent structural rearrangements.

Our genome-wide analysis of recurrent kataegis demonstrated that recurrent alterations are often in close proximity to recurrently amplified regions and established oncogenes in breast cancer. As such, this finding is consistent with the reported location of kataegis events in the vicinity of structural rearrangements^2–4,6^, although only 2% of rearrangements in the original BASIS study were reported to be associated with a kataegis loci^4^. Our observed localization of kataegis loci close to amplifications was particularly clear for the ER-positive tumor groups but also for ERnHER2p tumors with respect to the 17q12 locus (including ERBB2), although it should be noted that our studied ERnHER2p group is very small. In comparison, recurrent kataegis events were less frequent in TNBC and targeted different genomic loci (Fig. 5). Based on the used kataegis analysis tool, kataegis loci were most often annotated as distal intergenic or intronic, with only a small portion of loci annotated in the proximity of, or in, promoter regions. We found no support for kataegis loci targeting different genomic contexts or functional elements across clinical breast cancer subgroups. In agreement with D’ Antonio et al.^9^, we observed an enrichment of kataegis-associated SBSs (the SBS comprising the kataegis event) mapping to open chromatin regions and, to some extent, DHS regions, when compared to a complete SBS background in kataegis positive and negative TNBC and ERpHER2n tumors. However, in contrast to the report by D’ Antonio et al.^9^ we did not observe any consistent enrichment of kataegis-associated SBSs in transcription start sites or depletion in quiescent chromatin regions. As such, the actual functional impact of kataegis SBSs on topographical genome features or mRNA expression remains unclear.

Several limitations should be considered regarding the results presented in this study. The sample size for some clinical groups, particularly for the ERnHER2p and ERpHER2p groups, is small, which may impact findings. Moreover, as the BASIS cohort is not population representative, we acknowledge that frequency patterns may change if a similar analysis is performed in a representative ER and HER2 cohort. For analyses involving topographical features like chromatin states or regions of open chromatin, our study, along with other reported pan-cancer studies, is limited in that these features were mapped in samples unrelated to the studied cancers^21^. Another limitation is the lack of both complete transcriptional data and patient treatment and outcome data in the BASIS cohort, as well as an independent ER-positive validation cohort. These shortages preclude conclusions about the prognostic relevance of kataegis in ER-positive treatment groups, despite an apparent association with an aggressive tumor phenotype, stressing the need for further prognostically oriented investigations.

Taken together, in this study, we have focused on delineating kataegis in established breast cancer subgroups to provide a more nuanced understanding of its frequency and clinicopathological associations in primary breast cancer. Our findings show that in ERpHER2n disease, kataegis-positive tumors are associated with more aggressive disease characteristics, while in TNBC, the molecular implications and associations of kataegis, including its prognostic significance, remain less clear.

## Methods

### SCAN-B unselected population-based TNBC cohort

Based on the Sweden Cancerome Analysis Network—Breast (SCAN-B) study^22,23^, 235 TNBC patients diagnosed with primary invasive breast tumors and enrolled between 2010 and 2015 were included, originally reported in ref. ^13^. Specific patient inclusion and exclusion criteria for the SCAN-B cohort are reported in the original publication^13^, and patients in this cohort have previously been shown to be representative of the underlying breast cancer population of the healthcare region in which they were enrolled^13^. All tumors had curated WGS and RNA sequencing data (FPKM) available, as well as complete clinicopathological data, PAM50 subtypes, and TNBCtype^24^ subtypes (BL1, BL2, M, LAR)^13,25^. Clinicopathological and molecular characteristics of the 235 SCAN-B patients’ tumors are summarized in Table 1. Based on FPKM data, gene expression-based rank scores for eight biological metagenes in breast cancer originally defined by Fredlund et al.^14^ were calculated as described by Nacer et al.^26^. Pathology estimated proportions of tumor-infiltrating lymphocytes (TILs) on whole slide H&E sections were obtained from ref. ^27^. Proportions (exposure on tumor level) of SBS signatures (COSMIC v2) were taken as SigFit computed values from the study by Aine et al.^27^. Tumor driver alterations were obtained from deposited data associated with the study^13^.

### BASIS selected breast cancer cohort

The BASIS cohort comprises 560 patients of all clinical subtypes of breast cancer with curated WGS data reported by Nik-Zainal et al.^4^. BASIS is a selected cohort of breast cancers based on tissue samples from several European institutions collected over a large time span. Clinicopathological and molecular characteristics of BASIS patients’ tumors are summarized in Table 1. The BASIS cohort lacks complete treatment and patient follow-up data, limiting meaningful survival analyses. Of the 560 cases, 265 had available RNA-sequencing data (log2 transformed FPKM) and PAM50 subtypes as outlined in the original publication (using the AIMS PAM50 algorithm^28^). Of the 265 cases, 183 were ER-positive (ERp) (ERpHER2p or ERpHER2n). Based on FPKM data, gene expression-based rank scores for eight biological metagenes in breast cancer originally defined by Fredlund et al.^14^ were calculated as described by Nacer et al.^26^. Rank scores were computed individually for each tumor without any normalization or data centering (i.e., they represent single sample scores). BASIS TNBC cases with FPKM data were classified into the TNBCtype subtypes (BL1, BL2, M, LAR) using the online classification tool with default parameters^24^. Tumor driver alterations were obtained from deposited data associated with the study^4^.

### Kataegis analysis

SBSs were mapped to the hg19 genome built in the original studies. Analysis of kataegis was performed using the R *KataegisPortal* package (v1.0.3)^29^ with default settings, including a requirement of at least six consecutive SBSs with a maximum intermutation distance of 1000 bp. To map detected kataegis events to genes and functional elements in *KataegisPortal* the suggested packages from the vignette were used, including *BSgenome* (v1.66.3), *BSgenome.Hsapiens.UCSC.hg19* (v1.4.3), *ChIPseeker* (v1.34.1), and *TxDb.Hsapiens.UCSC.hg19.knownGene* (v3.2.2). Only kataegis events with confidence ≥1 in *KataegisPortal* were kept for further analyses. A positive binary kataegis status was inferred for each tumor if ≥1 event was recorded on chromosomes 1–23. Otherwise, the tumor was classified as kataegis negative. The involved SBSs for each kataegis event in each tumor were recorded for downstream analysis of enrichment in different genomic contexts and functional elements.

### Mapping of single base substitutions

SBSs in the BASIS and SCAN-B cohorts were mapped and annotated to different genomic contexts using open-access data. Briefly, each SBS was mapped to Assay for Transposase-Accessible Chromatin (ATAC) regions for breast cancer (obtained from ref. ^30^, defining regions of open chromatin), DNase I hypersensitive sites (DHS), different types of repetitive elements such as long interspersed nuclear elements (LINE), long terminal repeats (LTR), short interspersed nuclear elements (SINE), Simple_repeat, and Low_complexity regions (sourced from the UCSC genome browser tracks), exonic, intronic, and intergenic regions, CTCF-binding sites and regions of 18 different proposed chromatin states^31^.

### Statistical methods

All *p*-values reported are two-sided and were compared to a level of significance of 0.05 unless otherwise specified. Boxplot elements correspond to: (i) center line = median, (ii) box limits = upper and lower quartiles, (iii) whiskers = 1.5× interquartile range. FDR adjustment of driver gene analysis was performed using the p.adjust function in R. Differential gene expression analysis was performed using the significance analysis of microarray (SAM) method^32^. In the SCAN-B cohort, tumors with FPKM = 0 for a gene had their log2 FPKM value set to 0. In BASIS subgroups, only genes without any missing log2 FPKM data were used. Functional annotation enrichment analysis was performed using the *clusterProfiler* (v4.8.3) R package^33^. Input values were *t*-test *p*-values and log2-fold change values of all genes (processed for the SAM analysis). A multiple testing adjusted *p*-value < 0.05 using Benjamini–Hochberg (BH) correction was considered statistically significant. A gene list of 628 genes reported to be differentially expressed between breast cancers with and without kataegis^9^ were analyzed in subgroups using Student’s *t*-test on log2-transformed FPKM values similar to the SAM analysis.

### Survival analysis

Survival analyses were performed in R (v4.2.2) using the *survival* (v3.4.0) and *survminer* (v0.4.9) packages. Survival analyses were performed only in the SCAN-B TNBC cohort due to incomplete outcome data in BASIS. Distant recurrence-free interval (DRFI) defined according to the STEEP criteria^34^ was used as the primary endpoint for TNBC patients treated with standard-of-care adjuvant chemotherapy (FEC-based [combination of five fluorouracil, epirubicin, and cyclophosphamide] ± a taxane in 96% of cases) according to national guidelines. Details on patient inclusion and exclusion criteria, treatment details, endpoint definition, and CONSORT diagram relevant for the survival analysis of the SCAN-B TNBC cohort are available in^13^. Survival curves were estimated using the Kaplan–Meier method and compared using the log-rank test.

### Inclusion and ethics statement

This study is based on open-access data. All SCAN-B enrolled patients provided written informed consent prior to study inclusion as described in ref. ^13^. Ethical approval was given for the SCAN-B study (approval numbers 2009/658, 2010/383, 2012/58, 2013/459, 2015/277) by the Regional Ethical Review Board in Lund, Sweden, governed by the Swedish Ethical Review Authority, Box 2110, 750 02 Uppsala, Sweden. Patients in the BASIS cohort provided consent to research as outlined in the original publication^4^. All analyses performed complied with all relevant ethical regulations, including the Declaration of Helsinki.

### Supplementary information

Supplementary Information

Supplementary Table 1

Related Manuscript File

## Data Availability

Clinicopathological as well as somatic genetic data (variants) and RNA-sequencing data are freely available from publicly available repositories associated with the studies by Staaf et al.^13^ (SCAN-B cohort) and Nik-Zainal et al.^4^ (BASIS cohort). As such, the current study has not generated new sequencing or clinicopathological data.

## Code availability

All analyses were performed using open-source software such as Java and the R statistical language.
