# Supplementary material for: Kataegis in clinical and molecular subgroups of primary breast cancer
Source: NPJ Breast Cancer. 2024 Apr 24;10:32. doi: 10.1038/s41523-024-00640-8 (PMC11043427; doi:10.1038/s41523-024-00640-8)

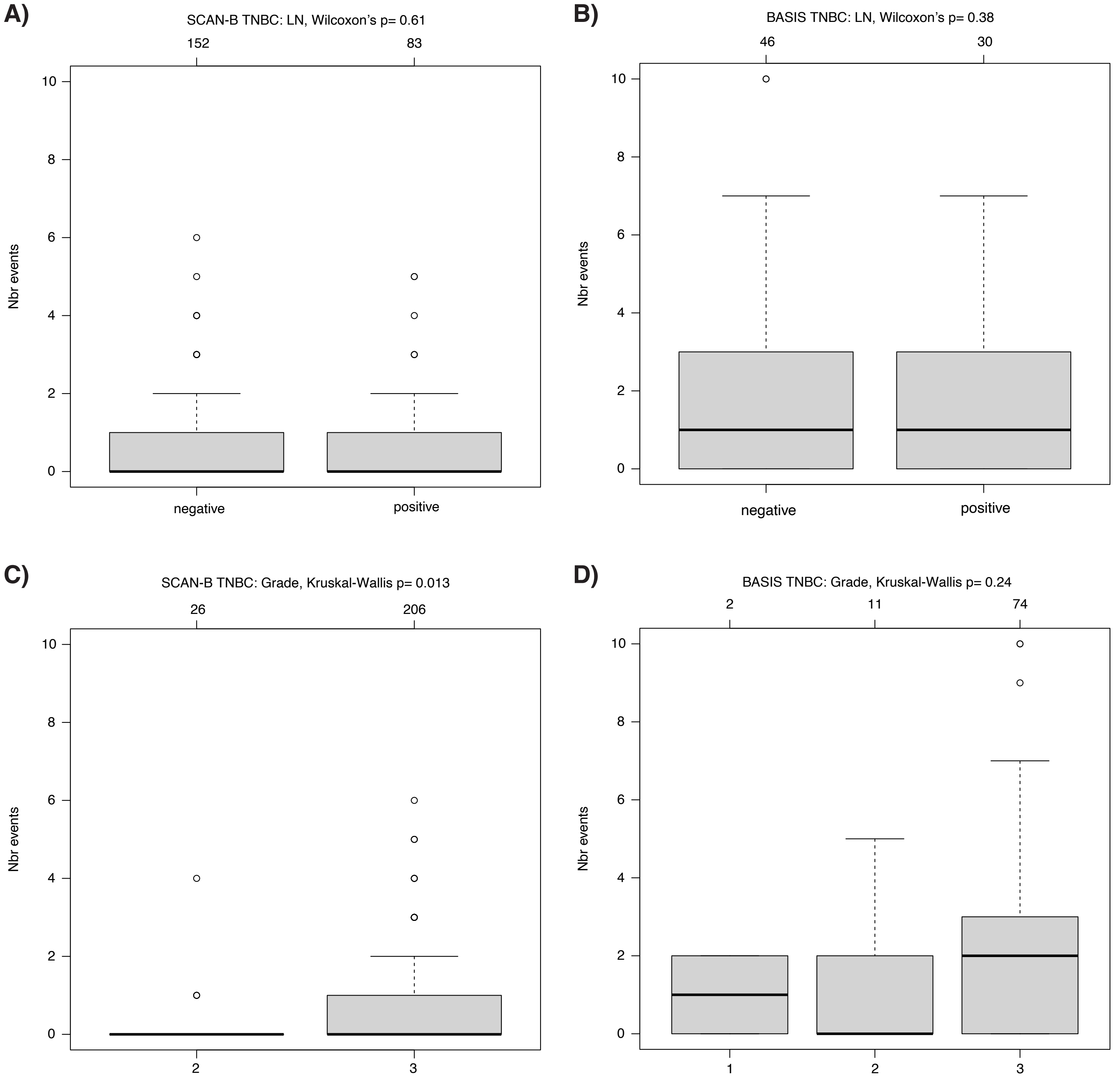

Supplementary Figure 1. Number of kataegis events in breast cancer subgroups and associations of binary kataegis status with copy number alteration metrics. In all panels the top axis shows the number of tumors per group. It should be noted that the BASIS cohort has missing values for some of the variables. **(A)** Number of kataegis events in SCAN-B TNBC tumors stratified by lymph node status. **(B)** Number of kataegis events in BASIS TNBC tumors stratified by lymph node status. **(C)** Number of kataegis events in SCAN-B TNBC tumors stratified by tumor grade. **(D)** Number of kataegis events in BASIS TNBC tumors stratified by tumor grade. **(E)** Tumor ploidy (by the ASCAT algorithm) in clinical subgroups stratified by binary kataegis status (positive: >=1 event in a tumor). Two ERpHER2n and one BASIS TNBC tumor are excluded due to failed analysis. **(F)** Distribution of the fraction of the genome altered by copy number gain or loss (CN-FGA) versus binary kataegis classification in clinical subgroups. Two ERpHER2n and one BASIS TNBC tumor are excluded due to failed analysis. A value of 0 means that no parts of the genome (chromosomes 1-22) are affected by somatic changes, whereas a value of 1 that the entire tumor genome is affected. **(G)** Distribution of fraction of the genome altered by LOH (LOH-FGA) versus binary kataegis classification in clinical subgroups. Two ERpHER2n and one BASIS TNBC tumor are excluded due to failed analysis. A value of 0 means that no parts of the genome (chromosomes 1-22) are affected by LOH, whereas a value of 1 that the entire tumor genome is affected. Tumor ploidy, CN-FGA, and LOH-FGA were obtained from the original studies. **(H)** Summary of p-values (-log10(p-value) as bar plots for patient age, tumor ploidy, CN-FGA, LOH-FGA, and tumor mutational burden (TMB) for different binary kataegis cut-off values in clinical subgroups. Bars >=1 corresponds to data shown in the main Figure 3. P-values are calculated using Wilcoxon's test. **(I)** Consistency of the differences in biological gene expression metagene rank scores for ERpHER2n tumors with respect to binary kataegis status (>=1 event) shown in main Figure 3, when compared to using >=2 (top row of panels) or >=3 (bottom row of panels) events as cut-off. P-values in panels were calculated using the Wilcoxons' test (2-groups) or the Kruskal-Wallis test (>2 groups). In A-D, the y-axis is truncated for illustrative purposes.

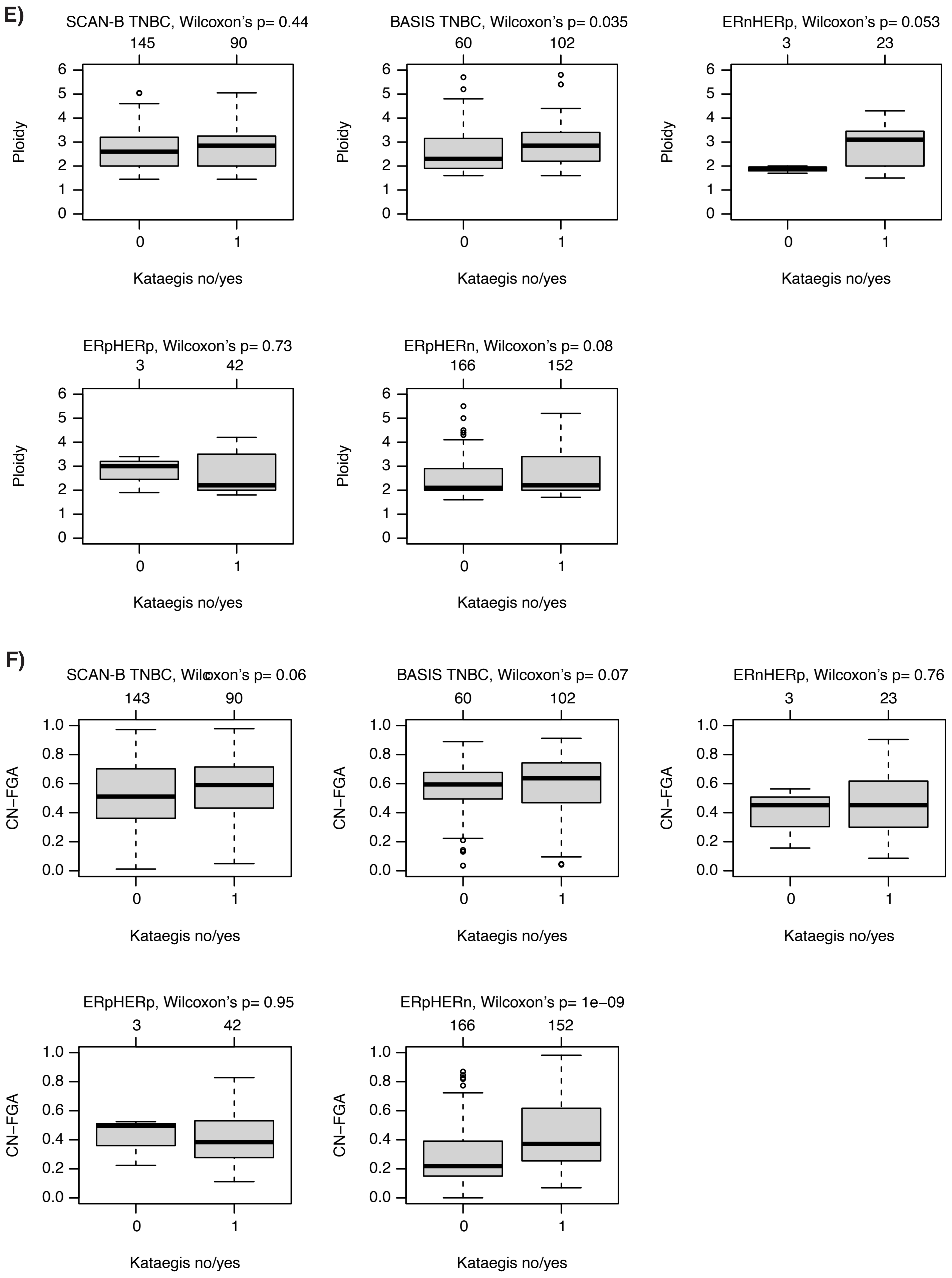
E)
SCAN-B TNBC, Wilcoxon's p= 0.44
145
90
BASIS TNBC, Wilcoxon's p= 0.035
60
102
ERnHERp, Wilcoxon's p= 0.053
3
23
ERpHERp, Wilcoxon's p= 0.73
3
42
ERpHERn, Wilcoxon's p= 0.08
166
152
Ploidy
Kataegis no/yes
F)
SCAN-B TNBC, Wilcoxon's p= 0.06
143
90
BASIS TNBC, Wilcoxon's p= 0.07
60
102
ERnHERp, Wilcoxon's p= 0.76
3
23
ERpHERp, Wilcoxon's p= 0.95
3
42
ERpHERn, Wilcoxon's p= 1e−09
166
152
CN−FGA
Kataegis no/yes

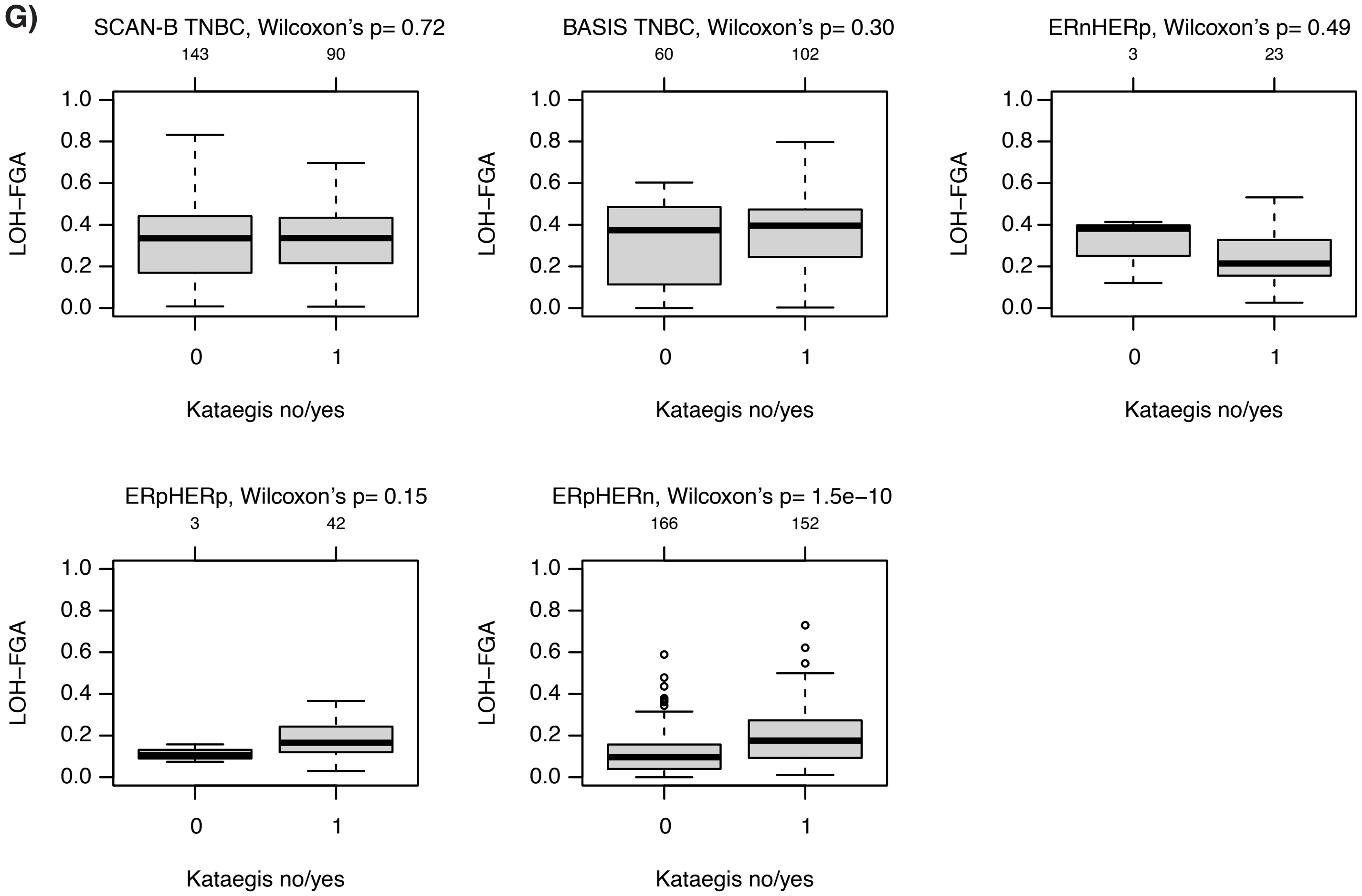
G)
SCAN-B TNBC, Wilcoxon's p= 0.72
143
90
BASIS TNBC, Wilcoxon's p= 0.30
60
102
ERnHERp, Wilcoxon's p= 0.49
3
23
ERpHERp, Wilcoxon's p= 0.15
3
42
ERpHERn, Wilcoxon's p= 1.5e−10
166
152
LOH−FGA
1.0
0.8
0.6
0.4
0.2
0.0
0
1
Kataegis no/yes

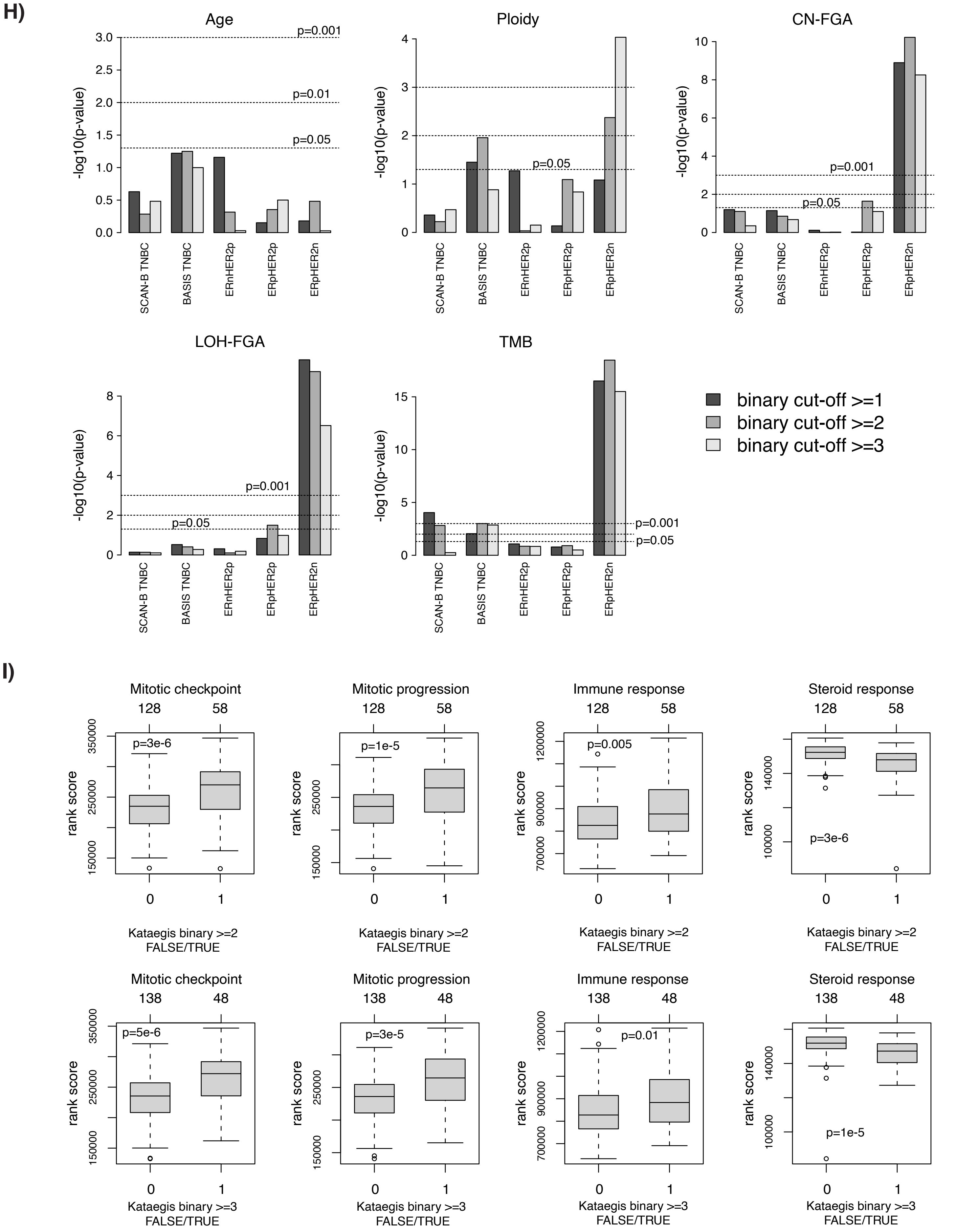

H)
Age
Ploidy
CN-FGA
LOH-FGA
TMB
-log10(p-value)
p=0.001
p=0.01
p=0.05
SCAN-B TNBC
BASIS TNBC
ERnHER2p
ERpHER2p
ERpHER2n
binary cut-off >=1
binary cut-off >=2
binary cut-off >=3
I)
Mitotic checkpoint
Mitotic progression
Immune response
Steroid response
128 58
138 48
rank score
p=3e-6
p=1e-5
p=0.005
p=5e-6
p=3e-5
p=0.01
Kataegis binary >=2
Kataegis binary >=3
FALSE/TRUE

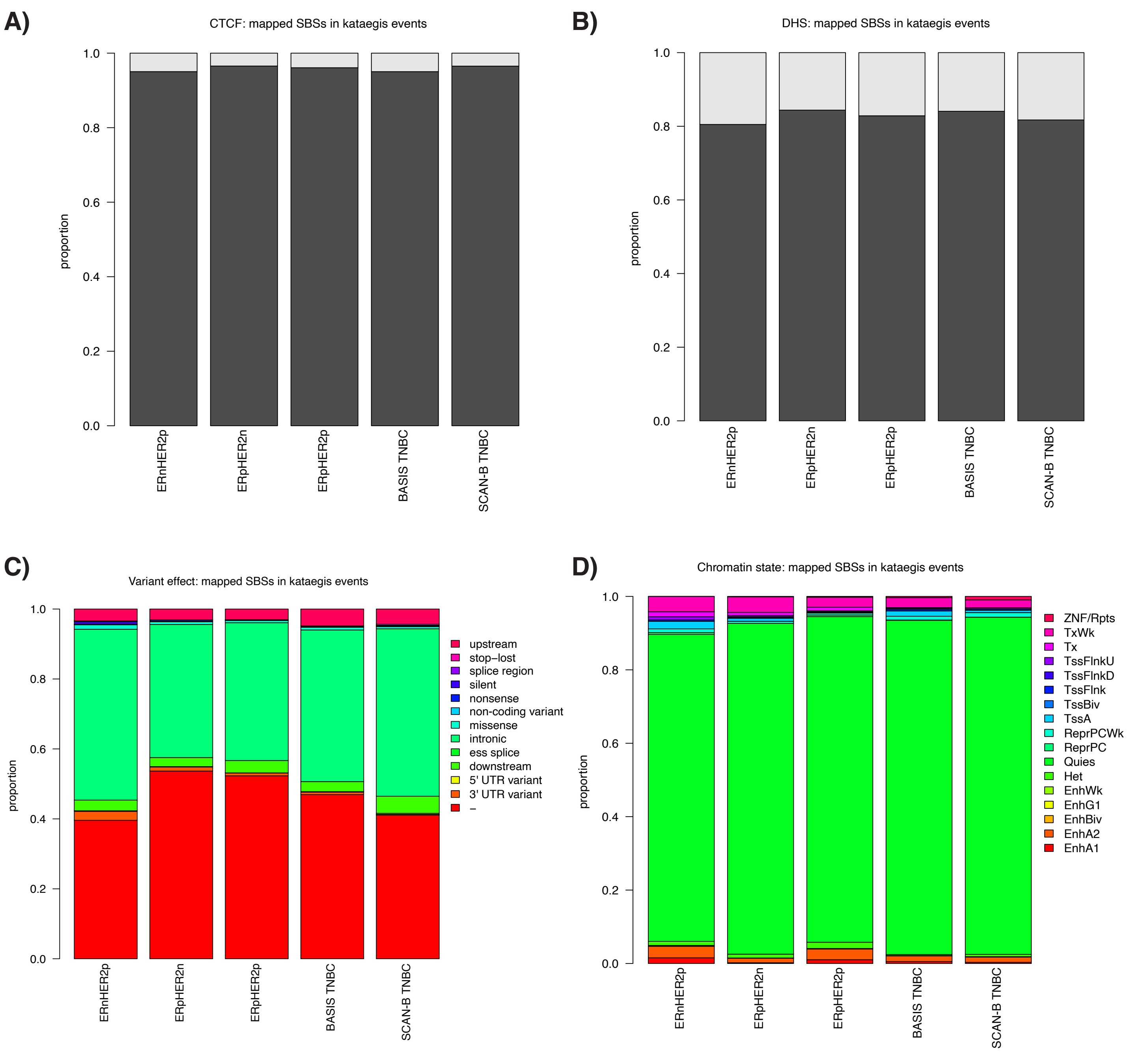

Supplementary Figure 2. Genomic contexts of kataegis in clinical breast cancer subgroups. In panels A-K the proportional bar for a subgroup represents the proportion of all kataegis associated SBSs in all kataegis affected tumors in that subgroup that are mapped (or not mapped) to a genomic context/feature/element. For variables with only two outcomes, the dark gray bar represents the not mapped fraction and the light gray bar represents the mapped fraction. **(A)** SBSs mapped to CTCF regions. **(B)** SBSs mapped to DHS regions. **(C)** SBSs mapped to SBS variant type. **(D)** SBSs mapped to different chromatin states. **(E)** SBSs mapped to genomic contexts. **(F)** SBSs mapped to repeat regions of any type. **(G)** SBSs mapped to LINE repeat regions. **(H)** SBSs mapped to SINE repeat regions. **(I)** SBSs mapped to simple repeat regions. **(J)** SBSs mapped to LTR repeat regions. **(K)** SBSs mapped to low complexity repeat regions.

E)

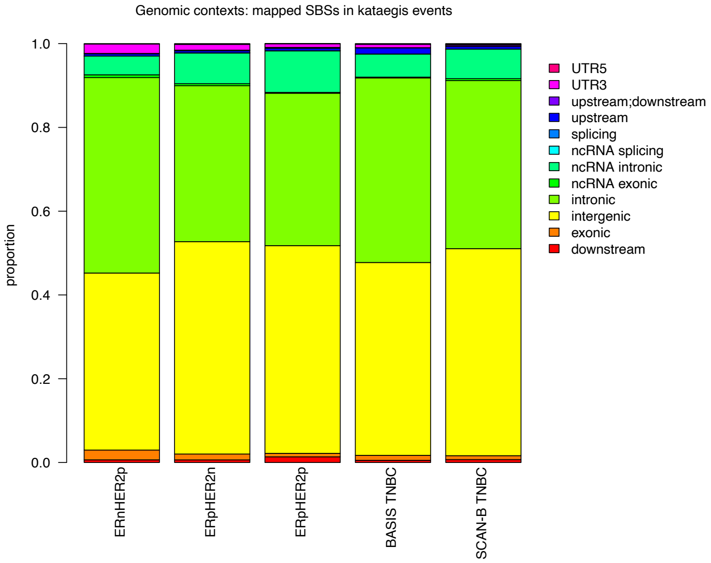
Genomic contexts: mapped SBSs in kataegis events
proportion
0.0
0.2
0.4
0.6
0.8
1.0
UTR5
UTR3
upstream;downstream
upstream
splicing
ncRNA splicing
ncRNA intronic
ncRNA exonic
intronic
intergenic
exonic
downstream
ERnHER2p
ERpHER2n
ERpHER2p
BASIS TNBC
SCAN-B TNBC

F)

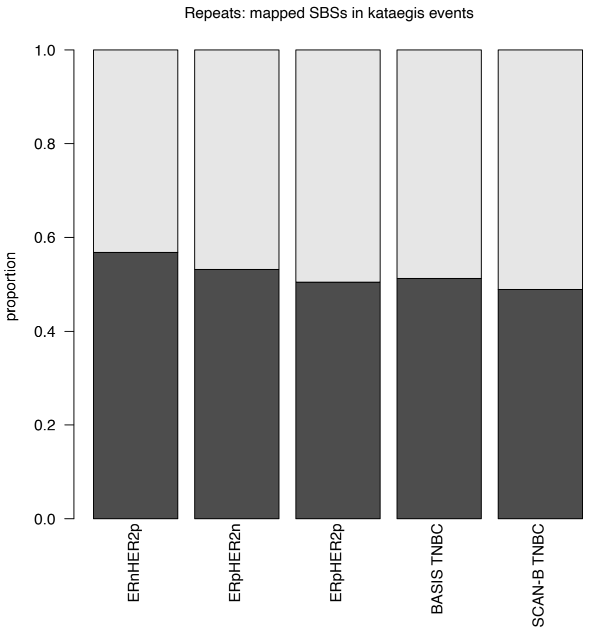
Repeats: mapped SBSs in kataegis events
proportion
0.0
0.2
0.4
0.6
0.8
1.0
ERnHER2p
ERpHER2n
ERpHER2p
BASIS TNBC
SCAN-B TNBC

G)

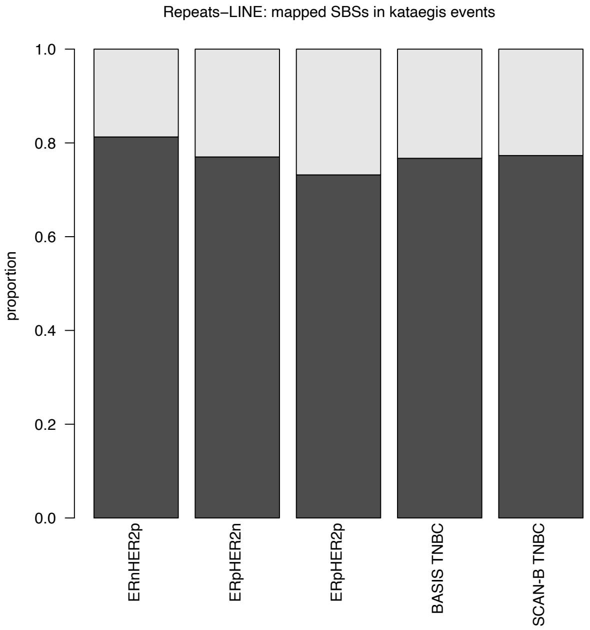
Repeats–LINE: mapped SBSs in kataegis events
proportion
0.0
0.2
0.4
0.6
0.8
1.0
ERnHER2p
ERpHER2n
ERpHER2p
BASIS TNBC
SCAN-B TNBC

H)

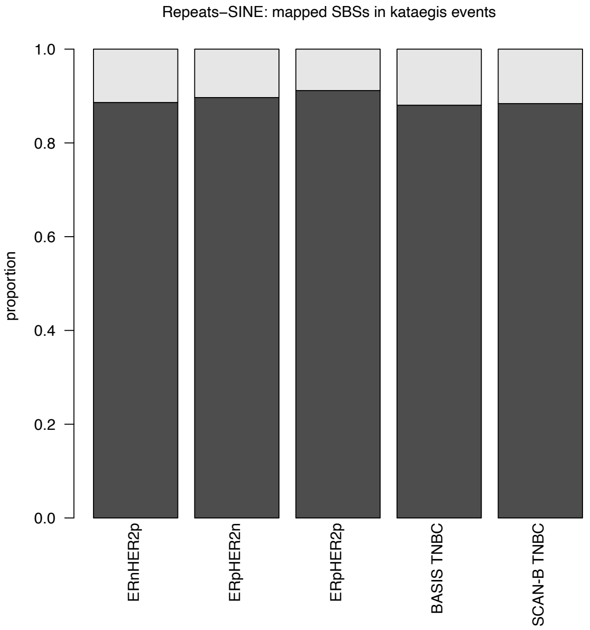
Repeats–SINE: mapped SBSs in kataegis events
proportion
0.0
0.2
0.4
0.6
0.8
1.0
ERnHER2p
ERpHER2n
ERpHER2p
BASIS TNBC
SCAN-B TNBC

I)

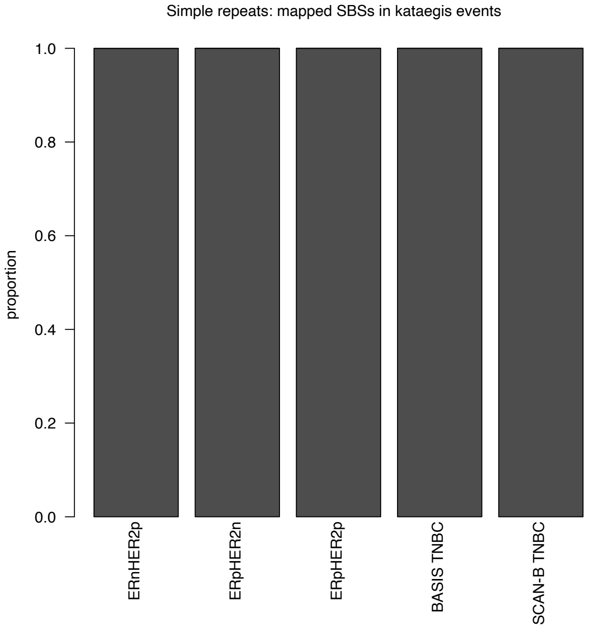
Simple repeats: mapped SBSs in kataegis events
proportion
0.0
0.2
0.4
0.6
0.8
1.0
ERnHER2p
ERpHER2n
ERpHER2p
BASIS TNBC
SCAN-B TNBC

J)

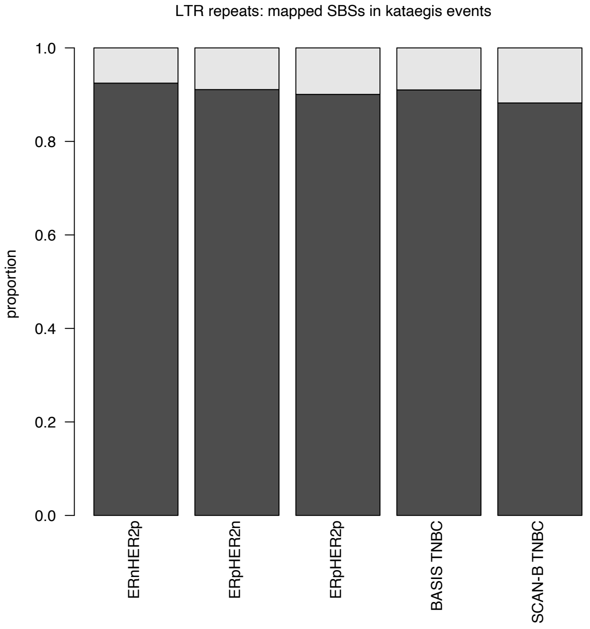
LTR repeats: mapped SBSs in kataegis events
proportion
0.0
0.2
0.4
0.6
0.8
1.0
ERnHER2p
ERpHER2n
ERpHER2p
BASIS TNBC
SCAN-B TNBC

K)

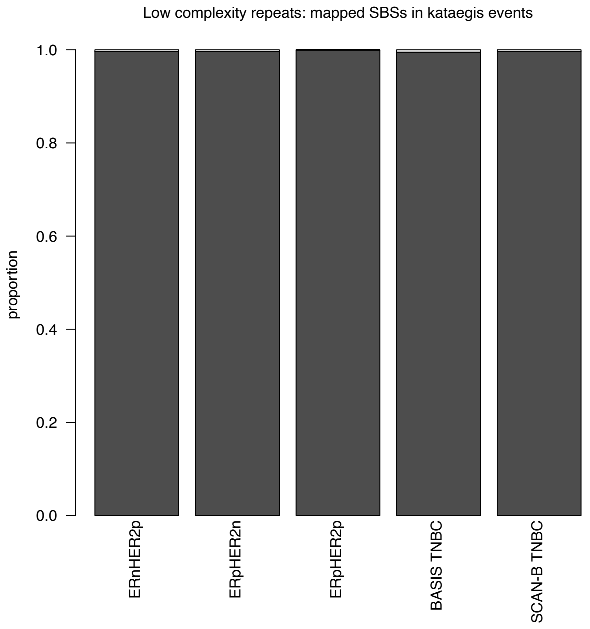
Low complexity repeats: mapped SBSs in kataegis events
proportion
1.0
0.8
0.6
0.4
0.2
0.0
ERnHER2p
ERpHER2n
ERpHER2p
BASIS TNBC
SCAN-B TNBC

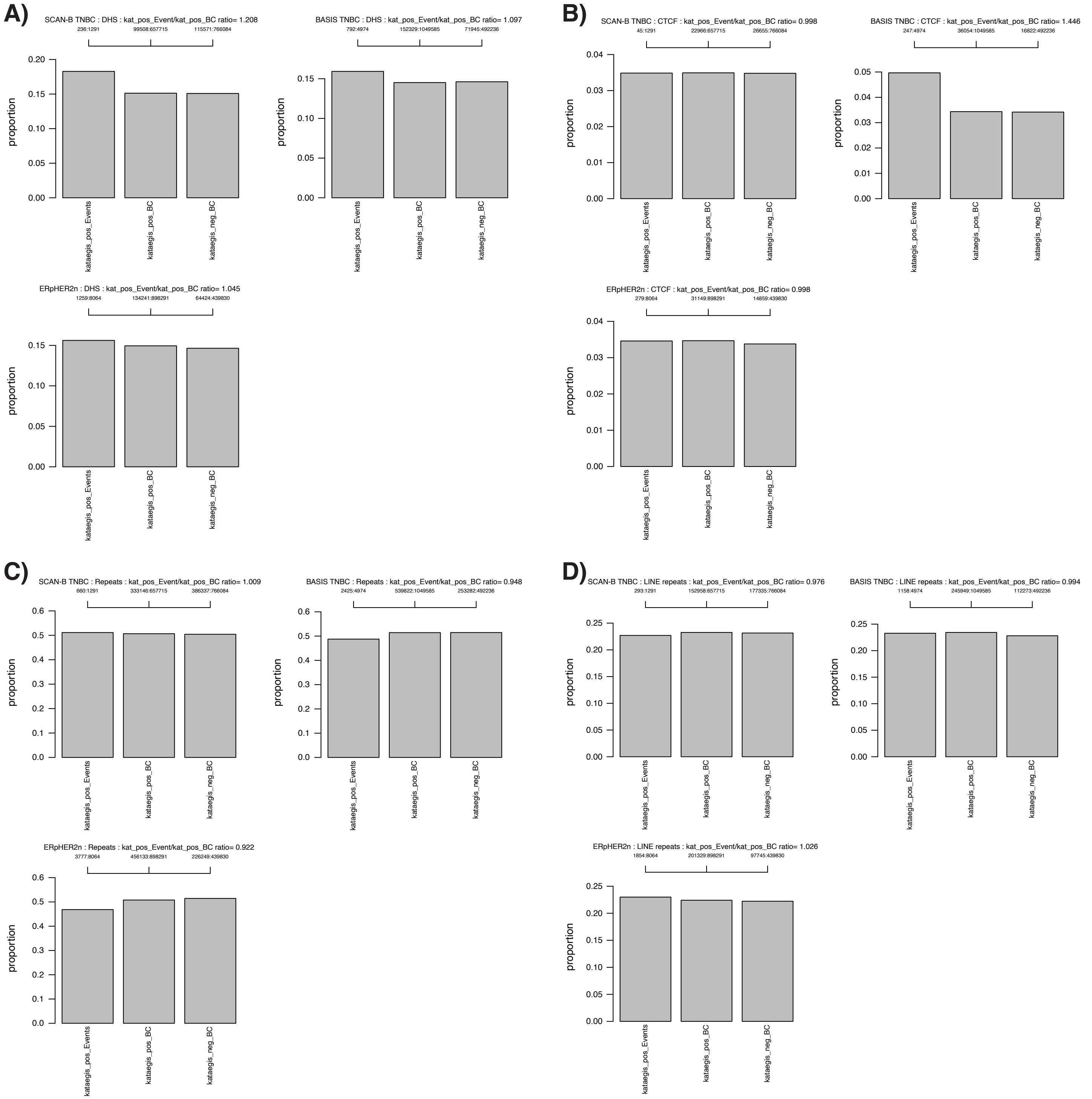

Supplementary Figure 3. Comparison of SBS proportions for kataegis associated variants versus proportions of all SBSs in kataegis positive and negative tumors in the TNBC and ERpHER2n subgroups. In panels A-N the left proportional bar for a clinical subgroup represents the proportion of all kataegis associated SBSs in all kataegis affected tumors in that subgroup that are mapped to a genomic context/feature/element (kataegis_pos_Events). The center bar bar corresponds to the same proportion calculated using all SBSs (kataegis_pos_BC) (thus including the kataegis SBSs) in kataegis positive tumors. The right bar corresponds to the same proportion calculated using all SBSs in kataegis negative tumors (kataegis_neg_BC). Thus, the difference between the left and center bar is that the left bar is based only on SBSs in kataegis loci for kataegis positive tumors, whereas the center bar is the corresponding value when including all SBSs detected in the kataegis positive tumors. The displayed ratio value for each comparison corresponds to the left bar (kataegis_pos_Events) divided by the center bar (kataegis_pos_BC). The top axis value for each bar outlines how many SBSs were in the mapped category (numerator), followed by the total number of SBSs in the group (denominator). **(A)** SBSs mapped to DHS regions. **(B)** SBSs mapped to CTCF regions. **(C)** SBSs mapped to repeat regions of any type. **(D)** SBSs mapped to LINE repeat regions. **(E)** SBSs mapped to SINE repeat regions. **(F)** SBSs mapped to simple repeat regions. **(G)** SBSs mapped to LTR repeat regions. **(H)** SBSs mapped to low complexity repeat regions. **(I)** SBSs mapped to intronic regions. **(J)** SBSs mapped to intergenic regions. **(K)** SBSs mapped to exonic regions. **(L)** SBSs mapped to quiescent (Quies) chromatin regions. **(M)** SBSs mapped to different types of transcription start site (Tss) elements. All Tss types from the chromatin state model were merged for the comparison. **(N)** SBSs mapped to different types of enhancer (Enh) elements. All enhancer subtypes from the chromatin state model were merged for the comparison.

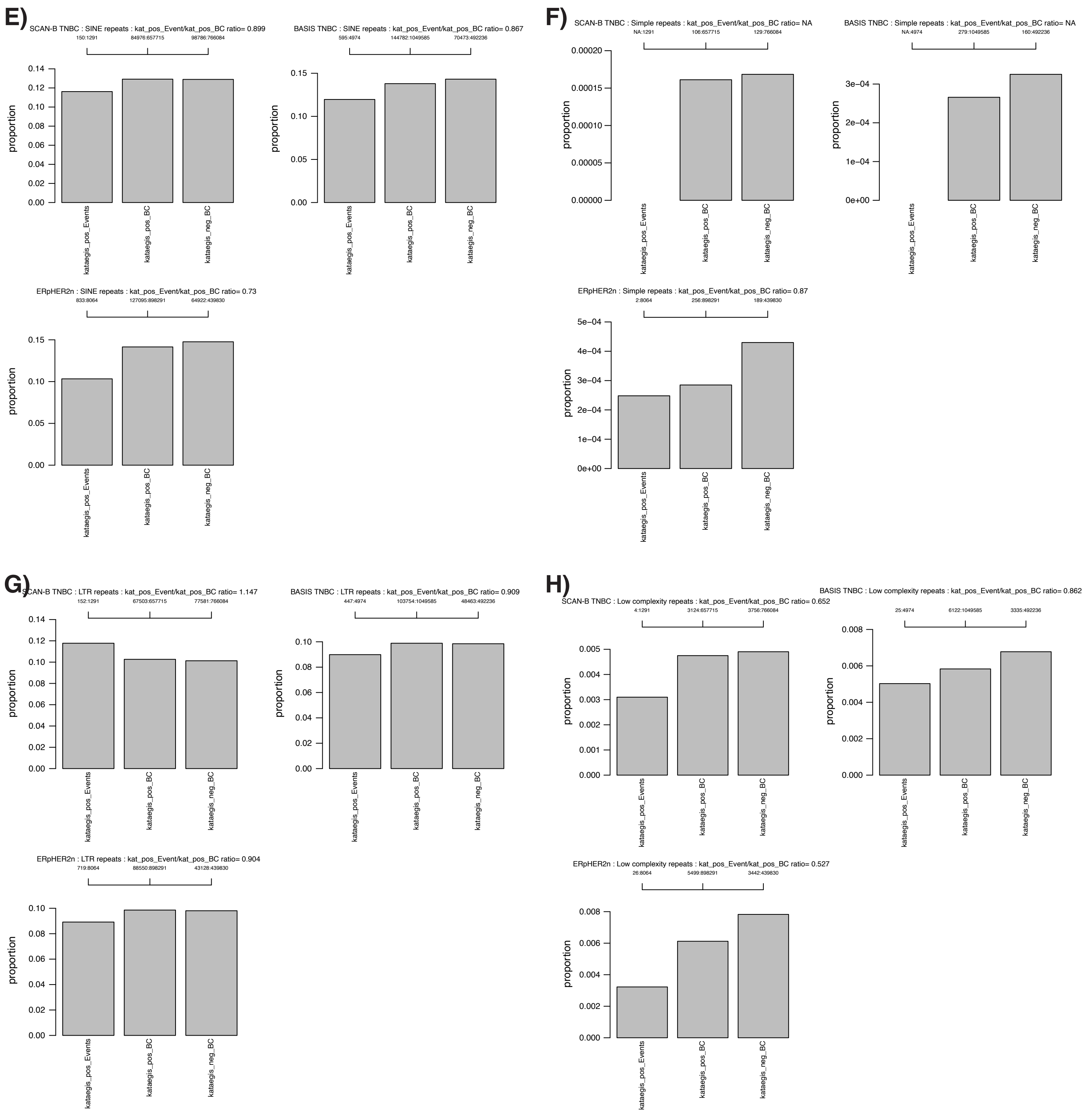
E)
SCAN-B TNBC : SINE repeats : kat_pos_Event/kat_pos_BC ratio= 0.899
150:1291
84976:657715
98786:766084
BASIS TNBC : SINE repeats : kat_pos_Event/kat_pos_BC ratio= 0.867
595:4974
144782:1049585
70473:492236
ERpHER2n : SINE repeats : kat_pos_Event/kat_pos_BC ratio= 0.73
833:8064
127095:898291
64922:439830
F)
SCAN-B TNBC : Simple repeats : kat_pos_Event/kat_pos_BC ratio= NA
NA:1291
106:657715
129:766084
BASIS TNBC : Simple repeats : kat_pos_Event/kat_pos_BC ratio= NA
NA:4974
279:1049585
160:492236
ERpHER2n : Simple repeats : kat_pos_Event/kat_pos_BC ratio= 0.87
2:8064
256:898291
189:439830
G)
SCAN-B TNBC : LTR repeats : kat_pos_Event/kat_pos_BC ratio= 1.147
152:1291
67503:657715
77581:766084
BASIS TNBC : LTR repeats : kat_pos_Event/kat_pos_BC ratio= 0.909
447:4974
103754:1049585
48463:492236
ERpHER2n : LTR repeats : kat_pos_Event/kat_pos_BC ratio= 0.904
719:8064
88550:898291
43128:439830
H)
SCAN-B TNBC : Low complexity repeats : kat_pos_Event/kat_pos_BC ratio= 0.652
4:1291
3124:657715
3756:766084
BASIS TNBC : Low complexity repeats : kat_pos_Event/kat_pos_BC ratio= 0.862
25:4974
6122:1049585
3335:492236
ERpHER2n : Low complexity repeats : kat_pos_Event/kat_pos_BC ratio= 0.527
26:8064
5499:898291
3442:439830
proportion
kataegis_pos_Events
kataegis_pos_BC
kataegis_neg_BC

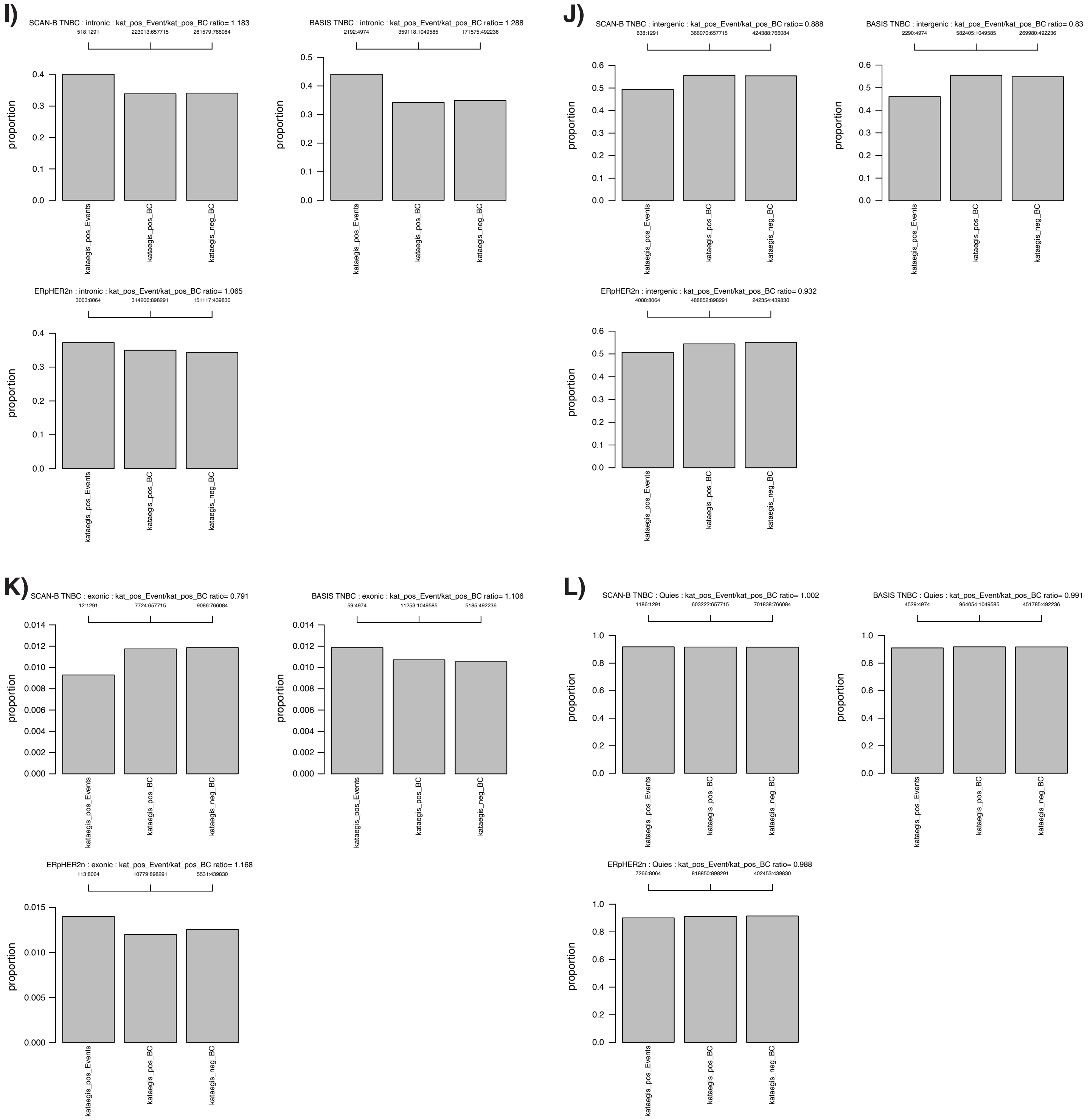
I)
SCAN-B TNBC : intronic : kat_pos_Event/kat_pos_BC ratio= 1.183
518:1291
223013:657715
261579:766084
BASIS TNBC : intronic : kat_pos_Event/kat_pos_BC ratio= 1.288
2192:4974
359118:1049585
171575:492236
ERpHER2n : intronic : kat_pos_Event/kat_pos_BC ratio= 1.065
3003:8064
314206:898291
151117:439830
J)
SCAN-B TNBC : intergenic : kat_pos_Event/kat_pos_BC ratio= 0.888
638:1291
366070:657715
424388:766084
BASIS TNBC : intergenic : kat_pos_Event/kat_pos_BC ratio= 0.83
2290:4974
582405:1049585
269980:492236
ERpHER2n : intergenic : kat_pos_Event/kat_pos_BC ratio= 0.932
4088:8064
488852:898291
242354:439830
K)
SCAN-B TNBC : exonic : kat_pos_Event/kat_pos_BC ratio= 0.791
12:1291
7724:657715
9086:766084
BASIS TNBC : exonic : kat_pos_Event/kat_pos_BC ratio= 1.106
59:4974
11253:1049585
5185:492236
ERpHER2n : exonic : kat_pos_Event/kat_pos_BC ratio= 1.168
113:8064
10779:898291
5531:439830
L)
SCAN-B TNBC : Quies : kat_pos_Event/kat_pos_BC ratio= 1.002
1186:1291
603222:657715
701838:766084
BASIS TNBC : Quies : kat_pos_Event/kat_pos_BC ratio= 0.991
4529:4974
964054:1049585
451785:492236
ERpHER2n : Quies : kat_pos_Event/kat_pos_BC ratio= 0.988
7266:8064
818850:898291
402453:439830
proportion
kataegis_pos_Events
kataegis_pos_BC
kataegis_neg_BC

M)

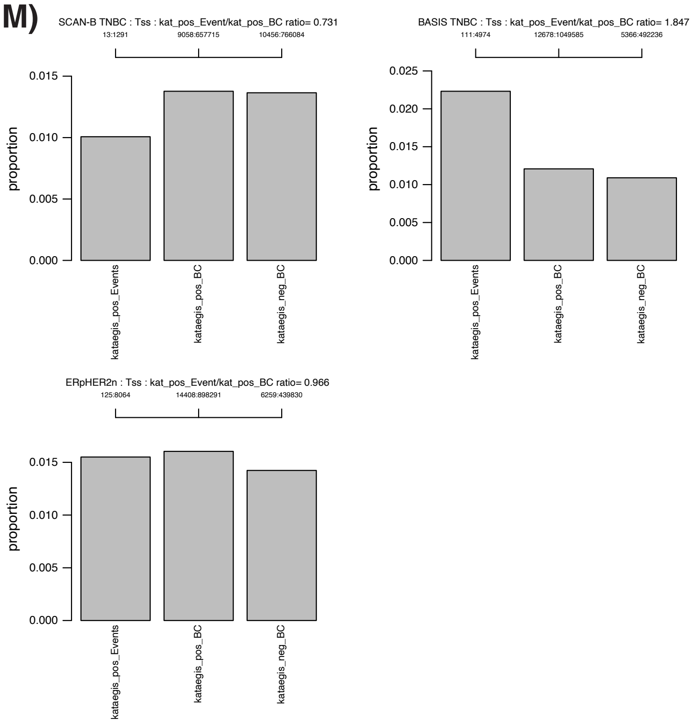
SCAN-B TNBC : Tss : kat_pos_Event/kat_pos_BC ratio= 0.731
13:1291
9058:657715
10456:766084
proportion
0.000
0.005
0.010
0.015
kataegis_pos_Events
kataegis_pos_BC
kataegis_neg_BC
BASIS TNBC : Tss : kat_pos_Event/kat_pos_BC ratio= 1.847
111:4974
12678:1049585
5366:492236
proportion
0.000
0.005
0.010
0.015
0.020
0.025
kataegis_pos_Events
kataegis_pos_BC
kataegis_neg_BC
ERpHER2n : Tss : kat_pos_Event/kat_pos_BC ratio= 0.966
125:8064
14408:898291
6259:439830
proportion
0.000
0.005
0.010
0.015
kataegis_pos_Events
kataegis_pos_BC
kataegis_neg_BC

N)

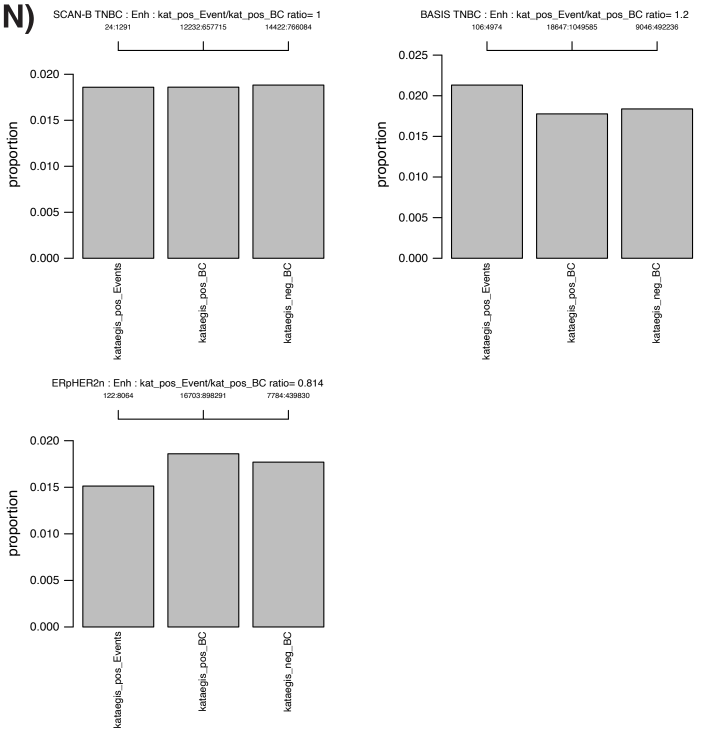
SCAN-B TNBC : Enh : kat_pos_Event/kat_pos_BC ratio= 1
24:1291
12232:657715
14422:766084
proportion
0.000
0.005
0.010
0.015
0.020
kataegis_pos_Events
kataegis_pos_BC
kataegis_neg_BC
BASIS TNBC : Enh : kat_pos_Event/kat_pos_BC ratio= 1.2
106:4974
18647:1049585
9046:492236
proportion
0.000
0.005
0.010
0.015
0.020
0.025
kataegis_pos_Events
kataegis_pos_BC
kataegis_neg_BC
ERpHER2n : Enh : kat_pos_Event/kat_pos_BC ratio= 0.814
122:8064
16703:898291
7784:439830
proportion
0.000
0.005
0.010
0.015
0.020
kataegis_pos_Events
kataegis_pos_BC
kataegis_neg_BC

A)

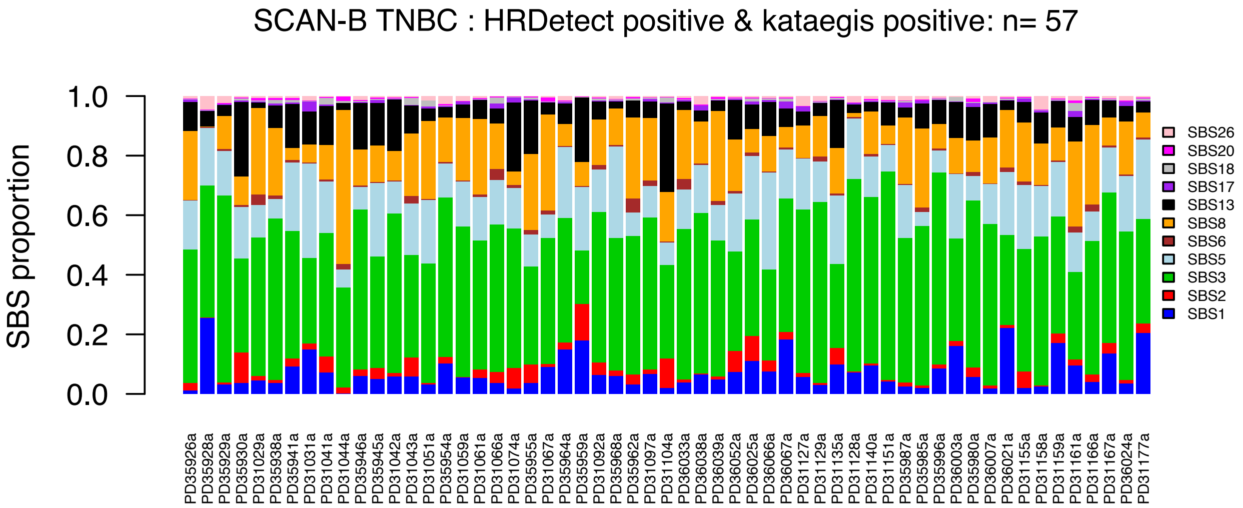

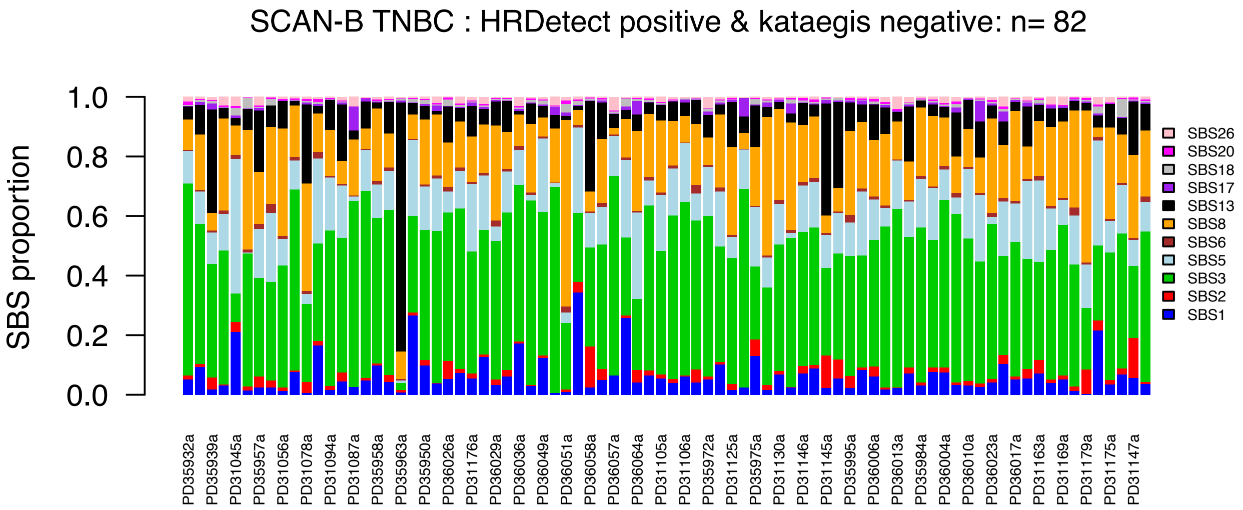

Supplementary Figure 4. SBS proportions in HRD-positive tumors stratified by kataegis status. SBS proportions for HRDetect positive tumors stratified by binary kataegis status (>=1 event) were investigated in **(A)** SCAN-B TNBC tumors, **(B)** BASIS TNBC tumors, and **(C)** ERpHER2n tumors. In A-C, black corresponds to SBS13 and red to SBS2, which are the main to APOBEC related SBS signatures. SBS proportions were obtained from original studies as described in the Material and Methods section.

B)

BASIS TNBC : HRDetect positive & kataegis positive: n= 50

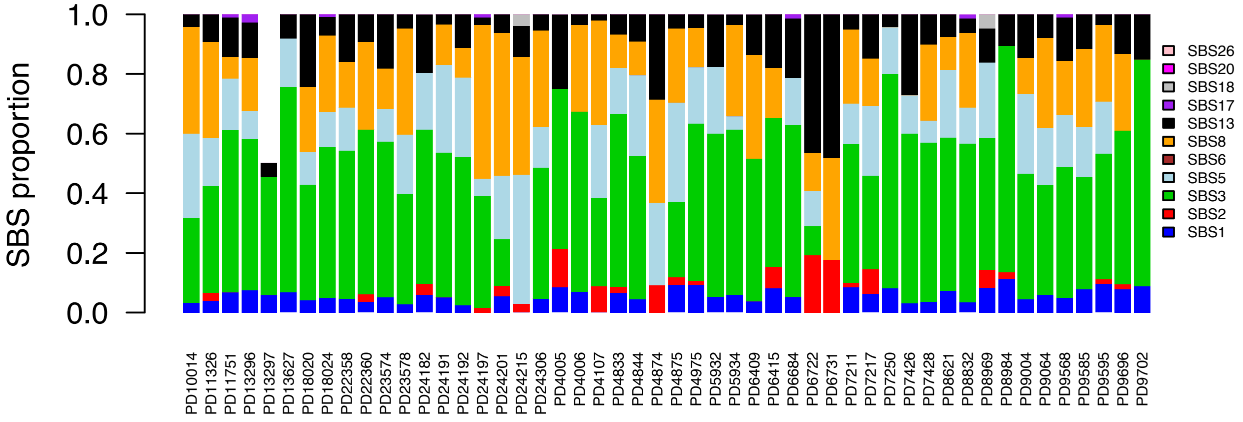

BASIS TNBC : HRDetect positive & kataegis negative: n= 29

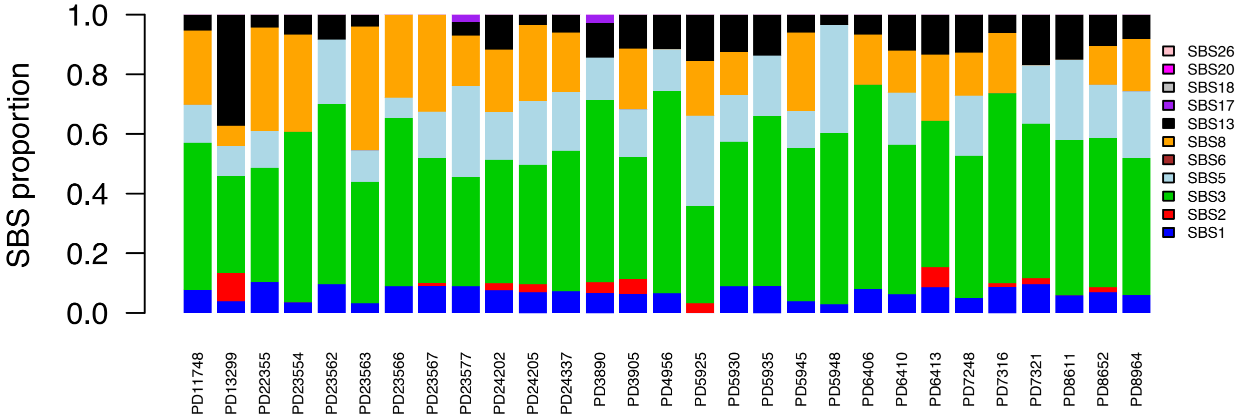

C)

ERpHER2n : HRDetect positive & kataegis positive: n= 17

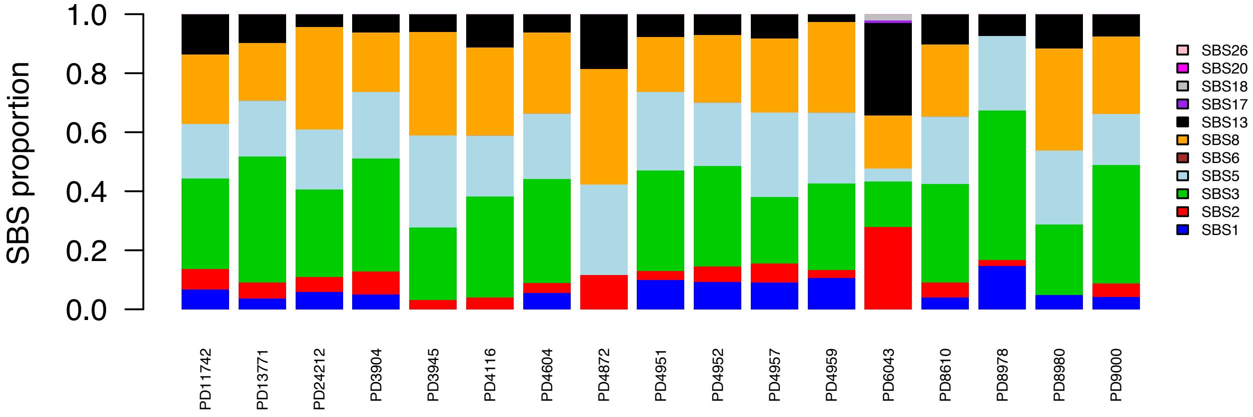

ERpHER2n : HRDetect positive & kataegis negative: n= 10

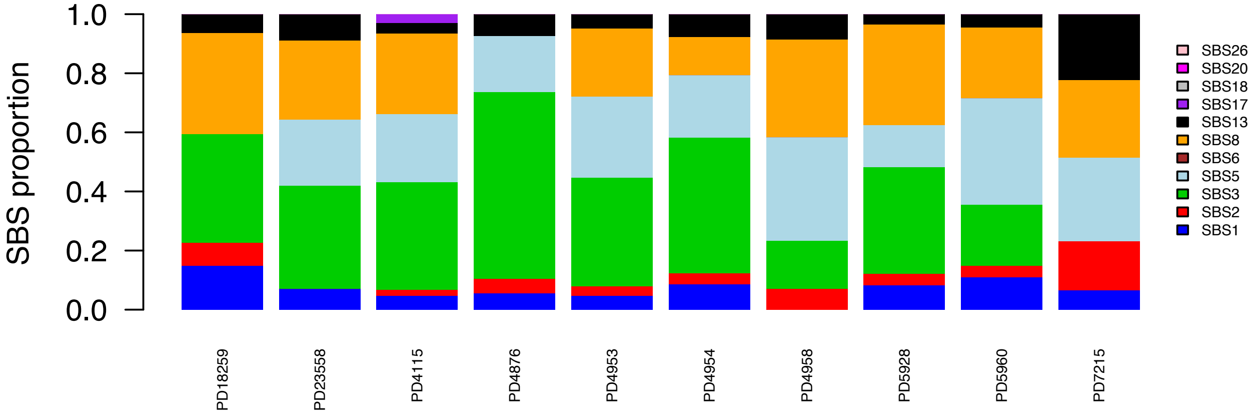

Supplement: Supplementary file 1 — Supplementary Information [file 41523_2024_640_MOESM1_ESM.pdf]
