# Supplementary material for: Kataegis in clinical and molecular subgroups of primary breast cancer
Source: NPJ Breast Cancer. 2024 Apr 24;10:32. doi: 10.1038/s41523-024-00640-8 (PMC11043427; doi:10.1038/s41523-024-00640-8)
Supplement: Supplementary file 3 — Related Manuscript File [file 41523_2024_640_MOESM3_ESM.pdf]

nature portfolio

Corresponding author(s): Johan Staaf

Last updated by author(s): Apr 6, 2024

Reporting Summary

Nature Portfolio wishes to improve the reproducibility of the work that we publish. This form provides structure for consistency and transparency in reporting. For further information on Nature Portfolio policies, see our Editorial Policies and the Editorial Policy Checklist.

Statistics

For all statistical analyses, confirm that the following items are present in the figure legend, table legend, main text, or Methods section.

n/a	Confirmed	
☐	☒	The exact sample size (*n*) for each experimental group/condition, given as a discrete number and unit of measurement
☒	☐	A statement on whether measurements were taken from distinct samples or whether the same sample was measured repeatedly
☐	☒	The statistical test(s) used AND whether they are one- or two-sided<br>*Only common tests should be described solely by name; describe more complex techniques in the Methods section.*
☒	☐	A description of all covariates tested
☐	☒	A description of any assumptions or corrections, such as tests of normality and adjustment for multiple comparisons
☐	☒	A full description of the statistical parameters including central tendency (e.g. means) or other basic estimates (e.g. regression coefficient) AND variation (e.g. standard deviation) or associated estimates of uncertainty (e.g. confidence intervals)
☐	☒	For null hypothesis testing, the test statistic (e.g. *F*, *t*, *r*) with confidence intervals, effect sizes, degrees of freedom and *P* value noted<br>*Give P values as exact values whenever suitable.*
☒	☐	For Bayesian analysis, information on the choice of priors and Markov chain Monte Carlo settings
☒	☐	For hierarchical and complex designs, identification of the appropriate level for tests and full reporting of outcomes
☐	☒	Estimates of effect sizes (e.g. Cohen's *d*, Pearson's *r*), indicating how they were calculated

Our web collection on statistics for biologists contains articles on many of the points above.

Software and code

Policy information about availability of computer code

Data collection

Somatic genetic data (variants) and RNA-sequencing data are available from publicly available repositories associated with the studies by Staaf et al. [13] (SCAN-B cohort) and Nik-Zainal et al. [4] (BASIS cohort). As such, the current study has not generated new sequencing data. Thus, we obtained pre-processed deposited data ready to use for analysis.

Data analysis

All analyses were performed using open-source software such as Java and the R statistical language.

SCAN-B unselected population-based TNBC cohort
Based on the Sweden Cancerome Analysis Network – Breast (SCAN-B) study [11, 12], 235 TNBC patients diagnosed with primary invasive breast tumors and enrolled between 2010 to 2015 were included, originally reported in [13]. Specific patient inclusion and exclusion criteria for the SCAN-B cohort are reported in the original publication [13], and patients in this cohort have previously been shown to be representative of the underlying breast cancer population of the healthcare region in which they were enrolled [13]. All tumors had curated WGS and RNA sequencing data (FPKM) available, as well as complete clinicopathological data, PAM50 subtypes, and TNBCtype [14] subtypes (BL1, BL2, M, LAR) [13, 15]. Clinicopathological and molecular characteristics for the 235 SCAN-B patients' tumors are summarized in Table 1. Based on FPKM data, gene expression-based rank scores for eight biological metagenes in breast cancer originally defined by Fredlund et al. [16] were calculated as described by Nacer et al. [17]. Pathology estimated proportions of tumor infiltrating lymphocytes (TILs) on whole slide H&E sections were obtained from [18]. Proportions (exposure on tumor level) of SBS signatures (COSMIC v2) were taken as SigFit computed values from the study by Aine et al. [18]. Tumor driver alterations were obtained from deposited data associated with the study [13].

BASIS selected breast cancer cohort
The BASIS cohort comprises 560 patients of all clinical subtypes of breast cancer with curated WGS data reported by Nik-Zainal et al. [4]. BASIS

is a selected cohort of breast cancers based on tissue samples from several European institutions collected over a large time span. Clinicopathological and molecular characteristics of BASIS patients' tumors are summarized in Table 1. The BASIS cohort lacks complete treatment and patient follow-up data, limiting meaningful survival analyses. Of the 560 cases, 265 had available RNA-sequencing data (log2 transformed FPKM) and PAM50 subtypes as outlined in the original publication (using the AIMS PAM50 algorithm [19]). Of the 265 cases, 183 were ER-positive (ERp) (ERpHER2p or ERpHER2n). Based on FPKM data, gene expression-based rank scores for eight biological metagenes in breast cancer originally defined by Fredlund et al. [16] were calculated as described by Nacer et al. [17]. Rank scores were computed individually for each tumor without any normalization or data centering (i.e., they represent single sample scores). BASIS TNBC cases with FPKM data were classified into the TNBCtype subtypes (BL1, BL2, M, LAR) using the online classification tool with default parameters [14]. Tumor driver alterations were obtained from deposited data associated with the study [4].

Kataegis analysis
SBSs were mapped to the hg19 genome build in the original studies. Analysis of kataegis was performed using the R KataegisPortal package (v1.0.3) [20] with default settings, including a requirement of at least six consecutive SBSs with a maximum intermutation distance of 1000bp. To map detected kataegis events to genes and functional elements in KataegisPortal the suggested packages from the vignette were used, including BSgenome (v1.66.3), BSgenome.Hsapiens.UCSC.hg19 (v1.4.3), ChIPseeker (v1.34.1), and TxDb.Hsapiens.UCSC.hg19.knownGene (v3.2.2). Only kataegis events with a confidence [illegible]1 in KataegisPortal were kept for further analyses. A positive binary kataegis status was inferred for each tumor if [illegible]1 event was recorded on chromosomes 1-23, otherwise the tumor was classified as kataegis negative. The involved SBSs for each kataegis event in each tumor were recorded for downstream analysis of enrichment in different genomic contexts and functional elements.

Mapping of single base substitutions
SBSs in the BASIS and SCAN-B cohorts were mapped and annotated to different genomic contexts using open access data. Briefly, each SBS was mapped to Assay for Transposase-Accessible Chromatin (ATAC) regions for breast cancer (obtained from [21], defining regions of open chromatin), DNase I hypersensitive sites (DHS), different types of repetitive elements such as LINE (Long Interspersed Nuclear Elements), LTR (Long Terminal Repeats), SINE (Short Interspersed Nuclear Elements), Simple_repeat, and Low_complexity regions (sourced from the UCSC genome browser tracks), exonic, intronic, and intergenic regions, CTCF-binding sites and regions of 18 different proposed chromatin states [22].

Statistical methods
All p-values reported are two-sided and were compared to a level of significance of 0.05 unless otherwise specified. Boxplot elements correspond to: (i) center line = median, (ii) box limits = upper and lower quartiles, (iii) whiskers = 1.5x interquartile range. FDR adjustment of driver gene analysis was performed using the p.adjust function in R. Differential gene expression analysis was performed using the Significance Analysis of Microarray (SAM) method [23]. In the SCAN-B cohort, tumors with FPKM=0 for a gene had their log2 FPKM value set to 0. In BASIS subgroups, only genes without any missing log2 FPKM data were used. Functional annotation enrichment analysis was performed using the clusterProfiler (v4.8.3) R package [24]. Input values were t-test p-values and log2 fold change values of all genes (processed as for the SAM analysis). A multiple testing adjusted p-value<0.05 using Benjamini-Hochberg (BH) correction was considered statistically significant. A gene list of 628 genes reported to be differentially expressed between breast cancers with and without kataegis [9], were analyzed in subgroups using Student's t-test on log2 transformed FPKM values similar to the SAM analysis.

Survival analysis
Survival analyses were performed in R (v4.2.2) using the survival (v3.4.0) and survminer (v0.4.9) packages. Survival analyses were performed only in the SCAN-B TNBC cohort, due to incomplete outcome data in BASIS. Distant recurrence-free interval (DRFI) defined according to the STEEP criteria [25] was used as the primary endpoint for TNBC patients treated with standard of care adjuvant chemotherapy (FEC-based [combination of 5 fluorouracil, epirubicin, and cyclophosphamide] ± a taxane in 96% of cases) according to national guidelines. Details on patient inclusion and exclusion criteria, treatment details, endpoint definition, and CONSORT diagram relevant for the survival analysis of the SCAN-B TNBC cohort are available in [13]. Survival curves were estimated using the Kaplan-Meier method and compared using the log-rank test.

For manuscripts utilizing custom algorithms or software that are central to the research but not yet described in published literature, software must be made available to editors and reviewers. We strongly encourage code deposition in a community repository (e.g. GitHub). See the Nature Portfolio guidelines for submitting code & software for further information.

Data

Policy information about availability of data

All manuscripts must include a data availability statement. This statement should provide the following information, where applicable:

- Accession codes, unique identifiers, or web links for publicly available datasets
- A description of any restrictions on data availability
- For clinical datasets or third party data, please ensure that the statement adheres to our policy

Somatic genetic data (variants) and RNA-sequencing data are available from publicly available repositories associated with the studies by Staaf et al. [13] (SCAN-B cohort) and Nik-Zainal et al. [4] (BASIS cohort). As such, the current study has not generated new sequencing data.

Research involving human participants, their data, or biological material

Policy information about studies with human participants or human data. See also policy information about sex, gender (identity/presentation), and sexual orientation and race, ethnicity and racism.

Reporting on sex and gender	Not relevant. All patients were women.
Reporting on race, ethnicity, or	Not relevant. No information on race, ethnicity or other socially relevant groupings was available for the SCAN-B and BASIS studies.

other socially relevant groupings	
Population characteristics	This study uses publicly deposited data associated with the SCAN-B (Staaf et al. Nature 2019) and BASIS (Nik-Zainal et al. Nature 2016) studies. Population details and characteristics are described in original studies. Table 1 in the current study outlines characteristics for key sample groups analyzed in this study.
Recruitment	Not relevant. This study uses publicly deposited data associated with the SCAN-B (Staaf et al. Nature 2019) and BASIS (Nik-Zainal et al. Nature 2016) studies. Recruitment is described in the original studies.
Ethics oversight	This study is based on open access data. All SCAN-B enrolled patients provided written informed consent prior to study inclusion as described in [13]. Ethical approval was given for the SCAN-B study (approval numbers 2009/658, 2010/383, 2012/58, 2013/459, 2015/277) by the Regional Ethical Review Board in Lund, Sweden, governed by the Swedish Ethical Review Authority, Box 2110, 750 02 Uppsala, Sweden. Patients in the BASIS cohort provided consent to research as outlined in the original publication [4].

Note that full information on the approval of the study protocol must also be provided in the manuscript.

Field-specific reporting

Please select the one below that is the best fit for your research. If you are not sure, read the appropriate sections before making your selection.

☒ Life sciences ☐ Behavioural & social sciences ☐ Ecological, evolutionary & environmental sciences

For a reference copy of the document with all sections, see nature.com/documents/nr-reporting-summary-flat.pdf

Life sciences study design

All studies must disclose on these points even when the disclosure is negative.

Sample size	Not applicable. This study uses publicly deposited data associated with the SCAN-B (Staaf et al. Nature 2019) and BASIS (Nik-Zainal et al. Nature 2016) studies. As such, sample sizes are fixed.
Data exclusions	This study uses publicly deposited data associated with the SCAN-B (Staaf et al. Nature 2019) and BASIS (Nik-Zainal et al. Nature 2016) studies. In the BASIS cohort 4 tumors were excluded based on incomplete ER, PR, HER2 status.
Replication	Not applicable. This study uses publicly deposited data associated with the SCAN-B (Staaf et al. Nature 2019) and BASIS (Nik-Zainal et al. Nature 2016) studies. No replication of WGS or RNAseq analyses were performed.
Randomization	Not applicable. This study uses publicly deposited data associated with the SCAN-B (Staaf et al. Nature 2019) and BASIS (Nik-Zainal et al. Nature 2016) studies.
Blinding	Not applicable. This study uses publicly deposited data associated with the SCAN-B (Staaf et al. Nature 2019) and BASIS (Nik-Zainal et al. Nature 2016) studies.

Reporting for specific materials, systems and methods

We require information from authors about some types of materials, experimental systems and methods used in many studies. Here, indicate whether each material, system or method listed is relevant to your study. If you are not sure if a list item applies to your research, read the appropriate section before selecting a response.

Materials & experimental systems

n/a	Involved in the study	
☒	☐	Antibodies
☒	☐	Eukaryotic cell lines
☒	☐	Palaeontology and archaeology
☒	☐	Animals and other organisms
☐	☒	Clinical data
☒	☐	Dual use research of concern
☒	☐	Plants

Methods

n/a	Involved in the study	
☒	☐	ChIP-seq
☒	☐	Flow cytometry
☒	☐	MRI-based neuroimaging

Clinical data

Policy information about clinical studies

All manuscripts should comply with the ICMJE guidelines for publication of clinical research and a completed CONSORT checklist must be included with all submissions.

Clinical trial registration	The SCAN-B study is a prospective observational study registered as ClinicalTrials.gov ID NCT02306096. As such it is not a phase 2 / 3 clinical trial. The BASIS study is not a registered trial.
Study protocol	The SCAN-B study can be accessed through ClinicalTrials.gov
Data collection	No data collection was performed for this study. Clinical data were obtained from the deposited data associated with the original SCAN-B and BASIS studies (see original studies for exact data).
Outcomes	Outcomes were obtained from the deposited data associated with the original SCAN-B and BASIS studies.

Plants

Seed stocks	NA
Novel plant genotypes	NA
Authentication	NA
